# Sulforaphane Synergizes With PD‐1 Blockade Through Activating CD8^+^ T Cells in Non–Small Cell Lung Cancer: Preclinical and Clinical Investigations

**DOI:** 10.1002/mco2.70688

**Published:** 2026-03-24

**Authors:** Jieyao Li, Jinyan Liu, Zheng Wang, Ming Zhao, Mingming You, Ziyi Fu, Caijuan Guo, Tengyue Zhang, Shasha Liu, Dongli Yue, Shuangning Yang, Yixin Li, Qun Gao, Yanfen Liu, Jianmin Huang, Liping Wang, Yi Zhang

**Affiliations:** ^1^ Biotherapy Center and Cancer Center The First Affiliated Hospital of Zhengzhou University Zhengzhou Henan China; ^2^ Department of Oncology The First Affiliated Hospital of Zhengzhou University Zhengzhou Henan China; ^3^ Zhongyuan Cell Therapy and Immunotherapy Laboratory Henan Academy of Innovations in Medical Science Zhengzhou Henan China; ^4^ Tianjian Laboratory of Advanced Biomedical Sciences Academy of Medical Sciences Zhengzhou University Zhengzhou Henan China; ^5^ School of Life Science Zhengzhou University Zhengzhou Henan China; ^6^ School of Public Health Zhengzhou University Zhengzhou Henan China

**Keywords:** anti‐PD‐1/PD‐L1 therapy, CD8^+^ T cells, non–small cell lung cancer, spurious progressions, sulforaphane

## Abstract

Anti‐PD‐1/PD‐L1 therapy has achieved promising success across several tumor types; however, its efficacy is still far from satisfactory in non–small cell lung cancer (NSCLC). Combining therapies have been attempted to synergize anti‐PD‐1/PD‐L1 therapy through activating antitumor response. Previously, we convinced the role of sulforaphane (SFN) in regulating tumor immune microenvironment (TME) to enhance antitumor response. Consistently, here we observed combining SFN with chemotherapy and anti‐PD‐1 therapy achieved the best tumor suppression versus other treatments in mouse models bearing Lewis lung carcinoma cells. Further, a clinical trial (KY‐2021‐0266) was performed, and the disease control and objective response rates were higher in the experimental group (SFN combined anti‐PD‐1 antibody and chemotherapy group, *n* = 30) compared with the control group (anti‐PD‐1 antibody combined chemotherapy group, *n* = 30) (100% vs. 93.3% and 86.7% vs. 60.0%, respectively). Moreover, the median progression‐free survival was longer (19 vs. 9.5 months, respectively) in the experimental group. After treatment, antitumor response was enriched, while CD8‐related function markers were elevated and myeloid‐derived suppressor cell/M2‐related markers were reduced in the experimental group. Two spurious progressions were observed in the experimental group. In conclusion, this synergistic effect suggests that SFN may be a promising immunosensitizer and a treatment option in NSCLC.

## Graphical abstract

SFN sensitizes PD‐1 blockade therapy in preclinical and clinical investigations, with no side effects. SFN elevates the antitumor response in peripheral and tumor microenvironment.

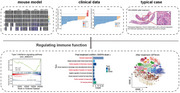

## 1 Introduction

For patients with non–small cell lung cancer (NSCLC) who lack genetic aberrations, platinum‐doublet chemotherapy has been the primary first‐line treatment for > 40 years; this option offers survival < 2 years for those with advanced or metastatic disease [1, 2]. Multicenter clinical trials have shown that combining immune checkpoint inhibitors targeting PD‐1 or PD‐L1 with chemotherapy could substantially improve progression‐free survival (PFS) and overall survival (OS) compared with chemotherapy alone in driver‐negative NSCLC, irrespective of PD‐L1 expression [3, 4, 5, 6]. In the CameL study [7], camrelizumab combined with chemotherapy resulted in an objective response rate (ORR) of 60.5%, median PFS of 11.3 months, and median OS of 27.9 months; these values were numerically superior to the historical data obtained after treatment with chemotherapy alone. Currently, these agents have become the cornerstone of first‐line therapy. However, some patients do not benefit from the combination of immunotherapy with chemotherapy [8]. Therefore, it is necessary to explore the optimal combination strategies to sensitize the combination of immunotherapy with chemotherapy in NSCLC.

The immune response is a major factor determining the efficacy of anti‐PD‐1 therapy [9, 10]. Hence, researchers have attempted to sensitize PD‐1/PD‐L1 pathway inhibitors using other therapies with immunomodulatory effects to enhance antitumor response [11, 12]. Nutrients play a role in cancer development and treatment [13]. Evidence suggested that vegetables from the Brassicaceae family containing isothiocyanates exhibit excellent activity in cancer prevention. Sulforaphane (SFN) was purified in 1992 and is the most commonly investigated isothiocyanate; it has been shown that SFN prevents and suppresses cancer [14]. Accumulating researches have been reported to convince the killing ability of SFN to cancer cells. SFN inhibited thioredoxin reductase 1 (TrxR1) and promoted reactive oxygen species (ROS) accumulation to induce glioblastoma (GBM) apoptosis [15]. In esophageal squamous cell carcinoma (ESCC), SFN suppressed cancer cells proliferation through activating NRF2‐related autophagy [16]. SFN induced osteosarcoma ferroptosis via modulating p62/SLC7A11 protein–protein interaction [17].

Besides, researchers revealed that SFN remarkably suppressed tumor growth in immunocompetent mice while only high concentration of SFN inhibited tumor volume in immunodeficient mice. Further, they found that SFN specifically modifies the cysteine residues of STAT1 protein, blocking the molecular mechanism of PD‐L1 transcriptional activation mediated by the IFN‐γ. This study reveals a new mode in which the active ingredient of SFN enhances the antitumor immune response by regulating immune checkpoints [18], suggesting SFN not only directly eliminating tumor cells, but also enhancing the antitumor response to indirectly killing tumor cells. Several studies have been reported to reveal the underlying mechanisms of SFN to modulate immune response. SFN regulated IFN‐β expression which in turn modulating cGAS‐STING signaling pathway to influence natural killer (NK) cells’ antitumor function [19]. And our previous research revealed that SFN treatment enhances antitumor response through reducing PD‐1 levels in chimeric antigen receptor‐T cells [20] and eliminating myeloid‐derived suppressor cells (MDSCs) [21]. These findings suggest that SFN influences antitumor response and, consequently, PD‐1 blockade.

Based on the preliminary research of SFN immunomodulating role, in this study, we examined whether SFN could sensitize chemotherapy and anti‐PD‐1 immunotherapy through preclinical and clinical investigations. We extracted SFN from broccoli seed and evaluated its role in sensitizing PD‐1 blockade in mouse models bearing Lewis lung carcinoma (LLC) cells. We observed that SFN supplementation significantly suppressed chemotherapy and anti‐PD‐1 therapy treated tumor growth. Flow cytometry was performed to detect the proportions of CD8^+^ T cells in blood; consistent with our previous study, SFN treatment elevated CD8^+^ T cells function. Subsequently, we conducted a clinical study to further assess the role of SFN. Patients with NSCLC were divided into control and experimental groups. In the experimental group, the disease control and ORRs were elevated as well as the median PFS. Corresponding to the animal data, SFN treatment significantly enhanced the antitumor response in NSCLC patients’ samples. Moreover, two spurious progressions, which unlike the reported earlier after treatment, these two patients appeared in the later treatment, were observed in the experimental group. Together with our previous research, this study further convinced the immunomodulating role of SFN and proved the synergistic effect of SFN with chemo‐ and immunotherapy, which emphasizes the importance of SFN as a promising immunosensitizer to enhance clinical treatment in NSCLC.

## 2 Results

### 2.1 SFN Enhanced Immune Response to Anti‐PD‐1 Antibody in Tumor‐Bearing Mice

**FIGURE 1:**
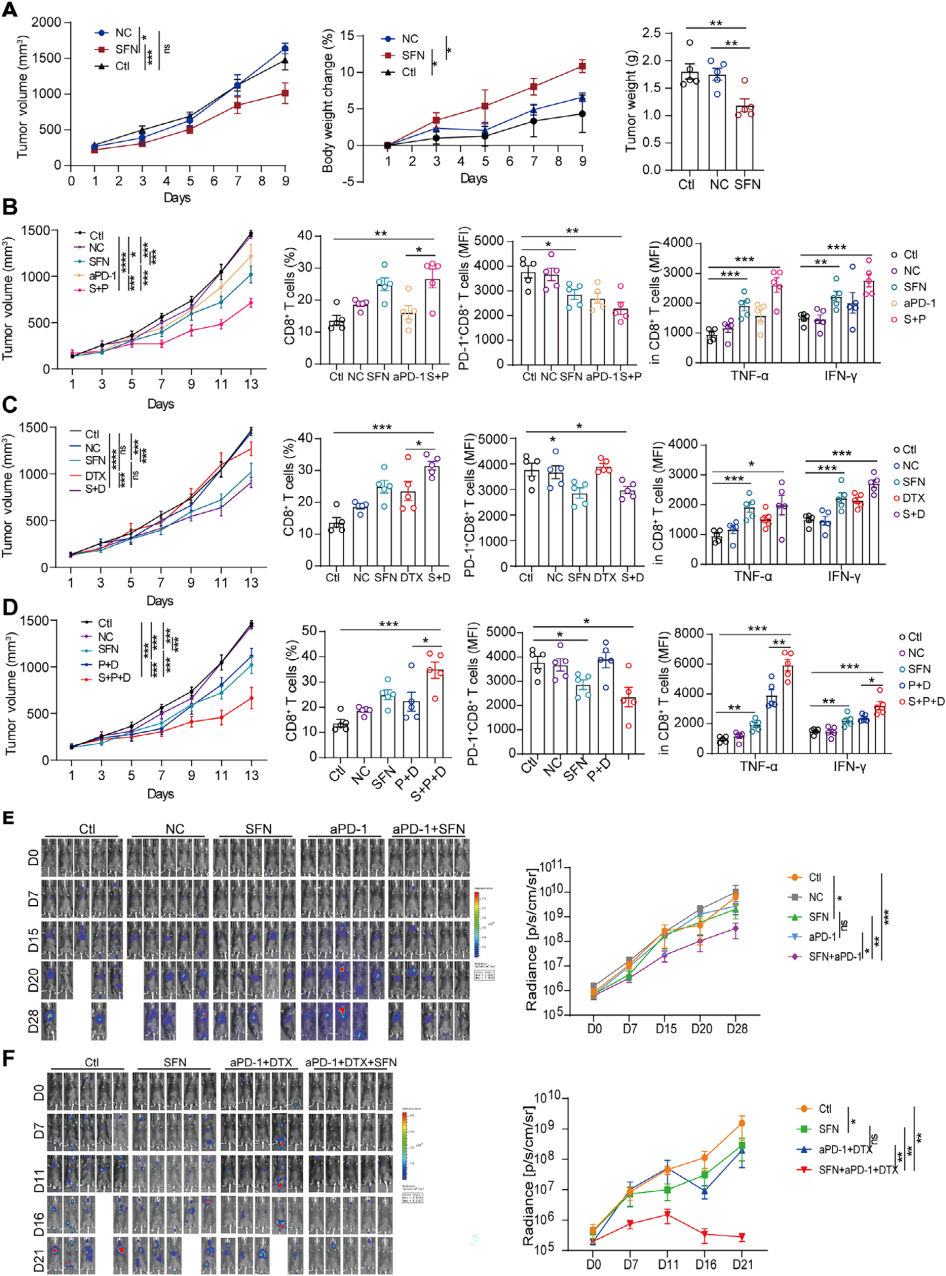
SFN enhanced a beneficial immune response of anti‐PD‐1 antibody in tumor‐bearing mice. The tumor growth curve, weight growth rate curve, and the tumor weight were compared between control group (Ctl), negative control (NC), and SFN group in subcutaneous tumor model. (A–D) The tumor volume growth curves of different groups were compared, as well as the proportion of CD8^+^ T cells, the expression of PD‐1 on tumor‐infiltrating CD8^+^ T cells, and the levels of TNF‐α and IFN‐γ secreted by tumor‐infiltrating CD8^+^ T cells. (E and F) In vivo BLI of lung metastasis developed after intravenous injection of Luc‐LLC cells. Representative images in the focused treatment group are shown, D0 refers to the day of treatment. Temporal BLI quantification (radiance, photons/s) based on the dosing timeline in thorax tumor burden. Data are mean ± SEM based on the indicated numbers of mice in various regimen groups (*n* = 5; ^*^ *p* < 0.05; ^**^ *p* < 0.01; ^***^ *p* < 0.001).

To determine whether SFN exerts an antitumor effect, the tumor‐bearing mouse model with subcutaneous tumor and lung metastatic tumor of tail vein was established. Tumor growth was significantly inhibited in the experimental group compared with the control and negative control groups (Figure 1A). Moreover, the most significant weight gain was observed in the experimental group (Figure 1A). Finally, tumor weight was significantly lower in the experimental group than the other groups (Figure 1A).

The ineffectiveness of anti‐PD‐1 antibody treatment may be partly attributed to the low proportion and impaired function of tumor‐infiltrating CD8^+^ T cells. To determine whether SFN could enhance the efficacy of anti‐PD‐1 antibody, we tested monotherapy and combination treatment regimens. In the SFN combined with anti‐PD‐1 antibody group, tumor growth was significantly inhibited (Figure 1B). Flow cytometry revealed that while the anti‐PD‐1 antibody alone reduced PD‐1 expression on CD8^+^ T cells, it did not enhance the proportion of CD8^+^ T cells in tumor tissues or their ability to secrete cytokines such as TNF‐α and IFN‐γ (Figure 1B). However, combination treatment with SFN increased the proportion of CD8^+^ T cells and the levels of cytokines they secreted (Figure 1B). Combination of docetaxel (DTX) with SFN significantly enhanced the treatment efficacy (Figure 1C). Flow cytometry analysis revealed that DTX did not elevate the proportion of CD8^+^ T cells within tumor tissues; nevertheless, it increased the expression of PD‐1 on CD8^+^ T cells (Figure 1C). Combination treatment with DTX and SFN increased the proportion of CD8^+^ T cells in tumor tissues and reduced the expression of PD‐1 on their surface. Moreover, the capacity of these cells to secrete cytokines (i.e., TNF‐α and IFN‐γ) also improved (Figure 1C).

Combination of immune checkpoint inhibitors with chemotherapy serves as the first‐line treatment for advanced NSCLC. To investigate whether the addition of SFN to anti‐PD‐1 antibody therapy combined with chemotherapy can further inhibit tumor growth, we administered SFN in combination with an anti‐PD‐1 antibody and DTX. The tumor volume in the anti‐PD‐1 antibody combined with DTX group increased slowly during the early stages of treatment and rapidly in the later stages. This observation indicated that the combination of anti‐PD‐1 antibody with DTX was unable to effectively inhibit tumor growth (Figure 1D). The addition of SFN inhibited tumor growth in the early stages and significantly suppressed the increase in tumor volume in the later stages (Figure 1D). Furthermore, there was a noticeable increase in the proportion of infiltrating CD8^+^ T cells within the tumor tissues, a decrease in PD‐1 expression on their surface (Figure 1D), and an enhanced ability to secrete cytokines (Figure 1D). These findings suggested that the addition of SFN to chemotherapy in combination with the anti‐PD‐1 antibody enhanced the cytotoxicity and antitumor effect of tumor‐infiltrating CD8^+^ T cells.

We further tested the antitumor effects of SFN alone and in combination with other agents in a mouse model of metastatic lung carcinoma. Combination of anti‐PD‐1 therapy with SFN showed synergy in the inhibition of tumor growth and increased survival compared with anti‐PD‐1 antibody monotherapy (Figure 1E). Furthermore, SFN combined with anti‐PD‐1 antibody and DTX reinforced the antitumor effects more than anti‐PD‐1 antibody plus DTX (Figure 1F). Moreover, three of five long‐term survivors in the three‐drug combination group displayed complete responses (Figure 1F).

### 2.2 SFN Increased the Antitumor Effect of Anti‐PD‐1 Therapy Combined With Chemotherapy in Patients With NSCLC

**FIGURE 2:**
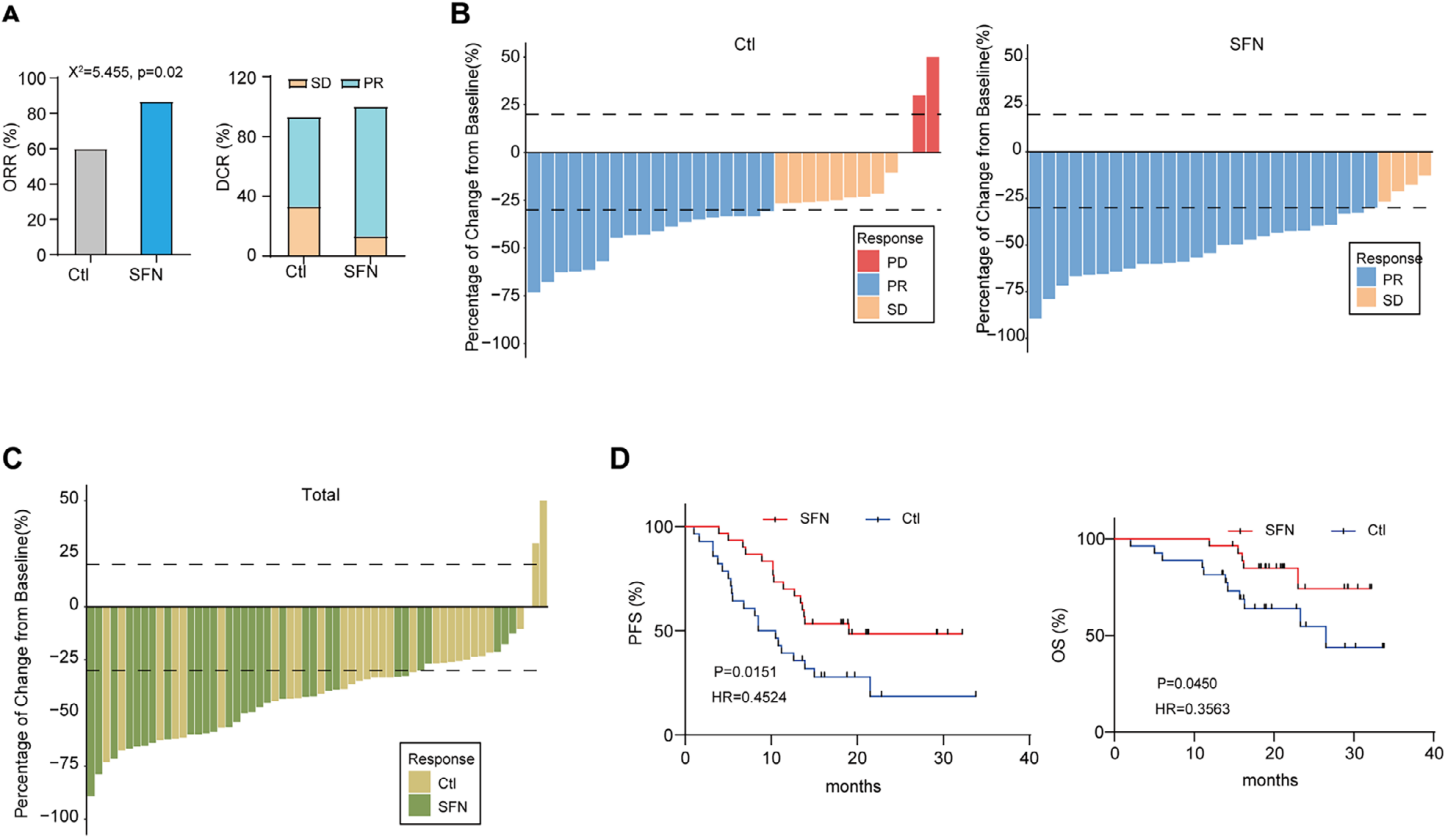
SFN increased the antitumor effect of anti‐PD‐1 antibody combined with chemotherapy in patients with NSCLC. (A) The ORR and DCR were evaluated between the two groups after matching. (B) Maximum percentage change of target lesions for each cohorts from baseline to disease progression, withdrawal from the study, or study completion. New lesions and inability to assess tumor size prescribed an increase of 20%. (C) Maximum percentage change of target lesions for 60 matched patients. (D) Kaplan–Meier curves of PFS and OS. The hazard ratio was estimated from a stratified Cox proportional hazards model. Comparisons between groups were analyzed using stratified one‐sided log‐rank test.

We tested whether the combination treatment was appropriate for real‐world clinical practice. Figure S1A shows the groups of patients with NSCLC included in this analysis. Baseline characteristics between control group and experimental group were comparable, allowing comparisons of efficacy and safety (Table 1 and Table S1). A careful examination of the tables revealed that after propensity score matching, many covariates that previously had *p*‐values less than 0.05 now showed *p*‐values greater than 0.05 (Table 1). This change in *p*‐values confirmed that our propensity score matching approach successfully balanced the distribution of baseline characteristics between the two groups (Figure S1B). Consequently, the potential influence of confounding factors was minimized, enabling a more reliable comparison of treatment effectiveness. After propensity score matching, prospective observations were conducted on 60 matched patients to evaluate the therapeutic effects of the two groups (Figure 2A–D and Table 2). There was one case of Stage II in each of the experimental group and the control group. Both cases were unable to undergo radical surgery immediately and therefore received neoadjuvant therapy prior to the surgery. Following three cycles of treatment, they both underwent radical surgery and recovered satisfactorily. The ORR was significantly higher in the experimental group than the control group (86.7% vs. 60.0%, respectively) (Figure 2A). Similarly, disease control rate (DCR) was 100% and 93.3%, respectively (Figure 2A), with a large reduction in tumor mass in the lungs of several patients in the experimental group (Figure 2B,C). Median PFS was 19.0 versus 9.5 months, respectively (hazard ratio [HR]: 0.45; 95% confidence interval [CI]: 0.23–0.88]; *p* = 0.015) (Figure 2D). The Kaplan–Meier was used to estimate median OS, with experimental groupnot reached while control group as 26.5 months, respectively (HR: 0.3; 95% CI: 0.13–0.96; *p* = 0.045) (Figure 2D).

**TABLE 1:** Patient demographics and baseline characteristics before and after matching.

Characteristics	Unmatched			Matched		
	Con, *N* = 45^a^	Exp, *N* = 35^a^	*p*‐value^b^	Con, *N* = 30^a^	Exp, *N* = 30^a^	*p*‐value^b^
Age			0.677			0.559
< 65	34 (76%)	25 (71%)		23 (77%)	21 (70%)	
≥ 65	11 (24%)	10 (29%)		7 (23%)	9 (30%)	
Sex			0.303			>0.999
Female	9 (20%)	4 (11%)		4 (13%)	4 (13%)	
Male	36 (80%)	31 (89%)		26 (87%)	26 (87%)	
Smoking			0.906			0.781
Yes	29 (64%)	23 (66%)		21 (70%)	20 (67%)	
No	16 (36%)	12 (34%)		9 (30%)	10 (33%)	
Pathology			0.222			0.605
Squamous	28 (62%)	17 (49%)		15 (50%)	17 (57%)	
Adenocarcinoma	17 (38%)	18 (51%)		15 (50%)	13 (43%)	
TNM			>0.999			0.692
2	1 (2%)	1 (3%)		1 (3%)	1 (3%)	
3	15 (33%)	11 (31%)		7 (23%)	11 (37%)	
4	29 (64%)	23 (66%)		22 (73%)	18 (60%)	
ECOG			0.896			0.766
0	34 (76%)	26 (74%)		23 (77%)	22 (73%)	
1	11 (24%)	9 (26%)		7 (23%)	8 (27%)	
PD‐L1			0.753			0.729
High	2 (4%)	3 (9%)		2 (7%)	3 (10%)	
Low	14 (31%)	9 (26%)		12 (40%)	9 (30%)	
Unknown	29 (64%)	23 (66%)		16 (53%)	18 (60%)	

^a^ *n* (%).

^b^ Pearson's chi‐squared test; Fisher's exact test.

**TABLE 2:** Effectiveness analysis for matched data.

	Unmatched		Matched	
Characteristics	Con	Exp	Con	Exp
*N*	*N* = 45	*N* = 35	*N* = 30	*N* = 30
Progression	33.00 (73.33%)	17.00 (48.57%)	23.00 (76.67%)	12.00 (40.00%)
Non‐progression	12.00 (26.67%)	18.00 (51.43%)	7.00 (23.33%)	18.00 (60.00%)
Unstratified response analysis				
Risk difference, RD (%) (95% CI)		−24.76 (−45.76 to −3.76)		−36.67 (−59.83 to −13.51)
Risk ratio, RR (95% CI)		0.66 (0.45–0.97)		0.52 (0.32–0.84)
Odds ratio, OR (95% CI)		0.34 (0.13–0.88)		0.20 (0.07–0.62)
*p*‐value (chi‐squared test)		0.0232		0.0040
*p*‐value (Fisher's exact test)		0.0357		0.0082

Our safety analysis revealed that the combination treatment including SFN did not significantly alter the levels of liver enzymes (i.e., alanine aminotransferase, aspartate aminotransferase, gamma‐glutamyltranspeptidase, total bilirubin, direct bilirubin, indirect bilirubin, and alkaline phosphatase); moreover, it did not affect kidney function markers, including blood urea nitrogen, creatinine, and glomerular filtration rate (Figure S1C–F). After treatment, gamma‐glutamyltranspeptidase was elevated in the control group, and direct bilirubin decreased in the experimental group (Figure S1E,F). The most common adverse events observed in both groups were consistent with the known profiles of anti‐PD‐1 antibody and chemotherapy (Table S2). However, the experimental group experienced a higher incidence of Grade 3–4 anemia, which was managed with appropriate medical interventions. These findings indicated that the addition of SFN to anti‐PD‐1 antibody therapy in combination with chemotherapy may offer a survival benefit for patients with advanced NSCLC. Further studies are warranted to confirm these results and to explore the potential mechanisms by which SFN enhances the efficacy of anti‐PD‐1 therapy.

### 2.3 SFN Treatment Enhanced Antitumor Peripheral Immune Response

**FIGURE 3:**
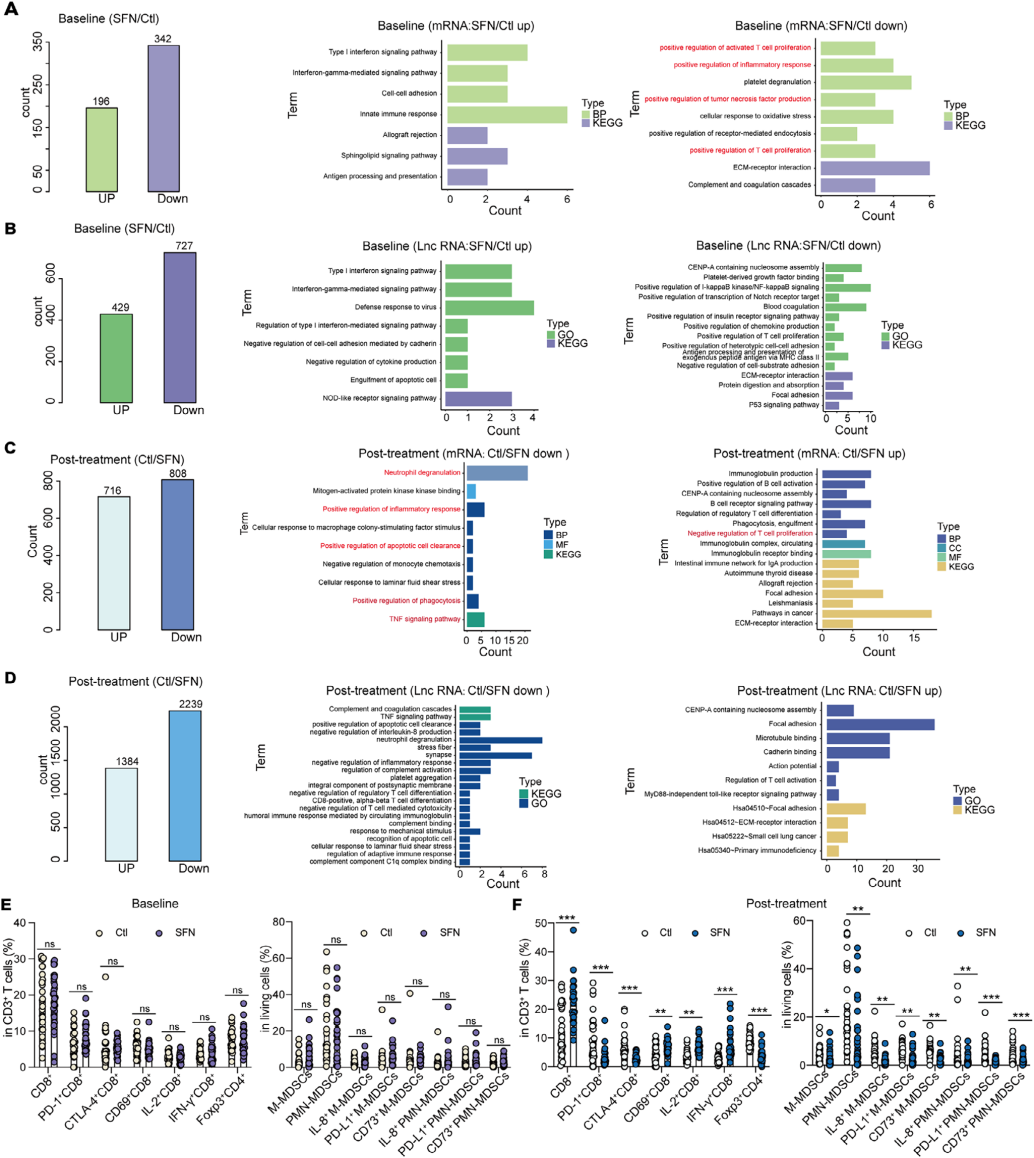
SFN treatment enhance the antitumor response in peripheral. (A) The different gene numbers of mRNA and GO/KEGG analysis were performed at the baseline in control and experimental groups. (B) The different gene numbers of lncRNA and GO/KEGG analysis were performed at the baseline in control and experimental group. (C) The different gene numbers of mRNA and GO/KEGG analysis were performed after treatment in control and experimental group. (D) The different gene numbers of lncRNA and GO/KEGG analysis were performed after treatment in control and experimental group. Flow cytometry was used to detect the CD8^+^ T cells and MDSCs at the baseline and after treatment in the control (E) and SFN (F) groups (ns, nonsignificant difference; ^*^ *p* < 0.05; ^**^ *p* < 0.01; ^***^ *p* < 0.001).

Multiple studies have reported the immunomodulatory role of SFN [20, 21]. We performed long noncoding RNA‐sequencing (lncRNA‐seq) to determine whether SFN regulates the peripheral immune response. Differential expression analysis of transcriptomic data revealed that genes were differentially regulated in the experimental group compared with the control group both at baseline and after treatment (Figure 3A–D and Figure S2A–D). At baseline, several pathways related to innate immune response, including Type I interferon signaling pathway, IFN‐γ‐mediated signaling pathway, innate immune response, etc., were enriched. While pathways related to adaptive immune response, including positive regulation of activated T cell proliferation, positive regulation of T cell proliferation, etc., were reduced in the experimental group (Figure 3A). The lncRNA‐seq yielded similar results (Figure 3B). The immune state differed between the two groups at baseline. Although the antigen presentation and interferon‐related pathways were enriched in the experimental group at baseline, the positive regulation of T cell function and proliferation was significantly reduced. After treatment, several pathways related to anti‐tumor response, including neutrophil degradation, positive regulation of inflammatory response, positive regulation of apoptotic cell clearance, TNF signaling pathway, etc., were downregulated, whereas pro‐tumor respomse related pathways, including the negative regulation of T cell proliferation, regulation of regulatory T (Treg) cell differentiation, etc., were enriched in the control group (Figure 3C). The lncRNA‐seq analysis yielded consistent results (Figure 3D), indicating that SFN treatment may activate T cell antitumor response and promote the clearance of apoptotic cells. In the control group, T cell chemotaxis, response to antigenic stimulus, T helper 17 cell differentiation, and the IL‐17 signaling pathway that are related to antitumor response were reduced post‐treatment (Figure S2A,B). In the experimental group, cytokine–cytokine receptor interaction, PI3K‐AKT signaling pathway, etc. were enriched after treatment (Figure S2C,D). Previously, we revealed that SFN could activate CD8^+^ T cells and induce MDSCs apoptosis [21]. Therefore, we preformed flow cytometry on peripheral blood mononuclear cells (PBMCs). At baseline, there was no difference in CD8^+^ T cells, FOXP3^+^CD4^+^ T cells, and MDSCs between the control and experimental groups (Figure 3E). SFN treatment significantly elevated the fraction of CD8^+^ T/CD69^+^CD8^+^ T cells, whereas it reduced those of PD‐1^+^/CTLA‐4^+^CD8^+^ T cells, IL‐8^+^/PD‐L1^+^/CD73^+^ MDSCs, and FOXP3^+^CD4^+^ T cells (Figure 3F). In the control group, the number of CD8^+^ T cells was reduced, those of PD‐1^+^ and CTLA‐4^+^CD8^+^ T cells, and FOXP3^+^CD4^+^ T cells were elevated, and that of MDSCs did not exhibit difference between baseline and after treatment (Figure S2E). Moreover, SFN treatment enhanced the fraction of CD8^+^ T/CD69^+^CD8^+^ T cells, whereas it reduced those of PD‐1^+^/CTLA‐4^+^CD8^+^ T cells, IL‐8^+^/PD‐L1^+^/CD73^+^ MDSCs, and FOXP3^+^CD4^+^ T cells compared with the baseline (Figure S2F). Collectively, these data indicated that SFN treatment could maintain and even enhance antitumor response in patients with NSCLC.

### 2.4 SFN Treatment Enhanced Antitumor Response in Tumor Sites Based on Spatially Resolved Single‐Cell Phenotypes Analysis

**FIGURE 4:**
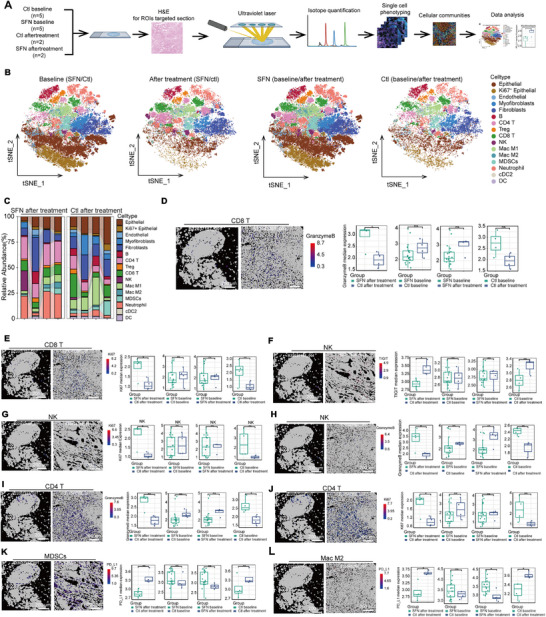
SFN treatment enhanced antitumor response in tumor sites based on spatially resolved single‐cell phenotypes analysis. (A) Experimental flowchart of the IMC analysis. (B) t‐distributed stochastic neighbor embedding (t‐SNE) was performed to define the cell types. (C) Proportions of cell types in control and experimental groups after SFN treatment. The levels of granzyme (D) and Ki67 (E) on CD8^+^ T cells were analyzed at the baseline, after treatment in control and experimental groups. The levels of TIGIT (F), Ki67 (G), and granzyme B (H) on NK cells were analyzed at the baseline, after treatment in control and experimental groups. The levels of granzyme (I) and Ki67 (J) on CD4^+^ T cells were analyzed at the baseline, after treatment in control and experimental groups. (K) The level of PD‐L1 on MDSCs was analyzed at the baseline, after treatment in control and experimental groups. (L) The level of PD‐L1 on M2 cells was analyzed at the baseline, after treatment in control and experimental groups (ns, nonsignificant difference; ^*^ *p* < 0.05; ^**^ *p* < 0.01; ^***^ *p* < 0.001).

In order to comprehensively illustrate the cellular heterogeneity and spatial organization of NSCLC tissue with or without SFN treatment, we utilized an IMC panel specific to lung histology (Figure 4A). We collected formalin‐fixed paraffin‐embedded tissues of patients from the control and experimental groups at baseline and after treatment. A 32‐plex antibody panel was used to identify immune cells, stromal/cancer cells, blood vessel (Figure S3A). We classified these cells into 16 types using a supervised lineage assignment method (Figure 4B and Figure S3B,C). Analysis revealed that the proportions of epithelial, Ki67^+^ epithelial, and neutrophils were higher at baseline in the experimental group (Figure S3D–H and Figure S4A), while they were reduced after treatment (Figure 4C and Figure S4B). SFN treatment reduced the proportions of epithelial and Ki67^+^ epithelial, whereas it elevated those CD4^+^ and CD8^+^ T cells (Figure S3D and Figure S4C). No differences were observed in the control group between baseline and after treatment (Figure S3D and Figure S4D).

We further analyzed the function‐related markers of immune cells. Compared with control, SFN treatment significantly elevated granzyme B and Ki67 levels on CD8^+^ T cells (Figure 4D,E). For NK cells, SFN treatment enhanced the granzyme B and Ki67 levels, whereas it reduced TIGIT levels (Figure 4F–H). Similarly, SFN treatment significantly elevated granzyme B and Ki67 levels on CD4^+^ T cells (Figure 4I,J). In addition, SFN treatment reduced PD‐L1 expression on MDSCs and M2 cells compared with baseline (Figure 4K,L). Taken together, these data imply that SFN treatment enhanced antitumor response in the NSCLC tumor microenvironment ignorance the imbalanced baseline.

### 2.5 SFN Treatment Suppressed Myofibroblast Function and Enhanced Immune Cell Interplay in the Tumor Microenvironment

**FIGURE 5:**
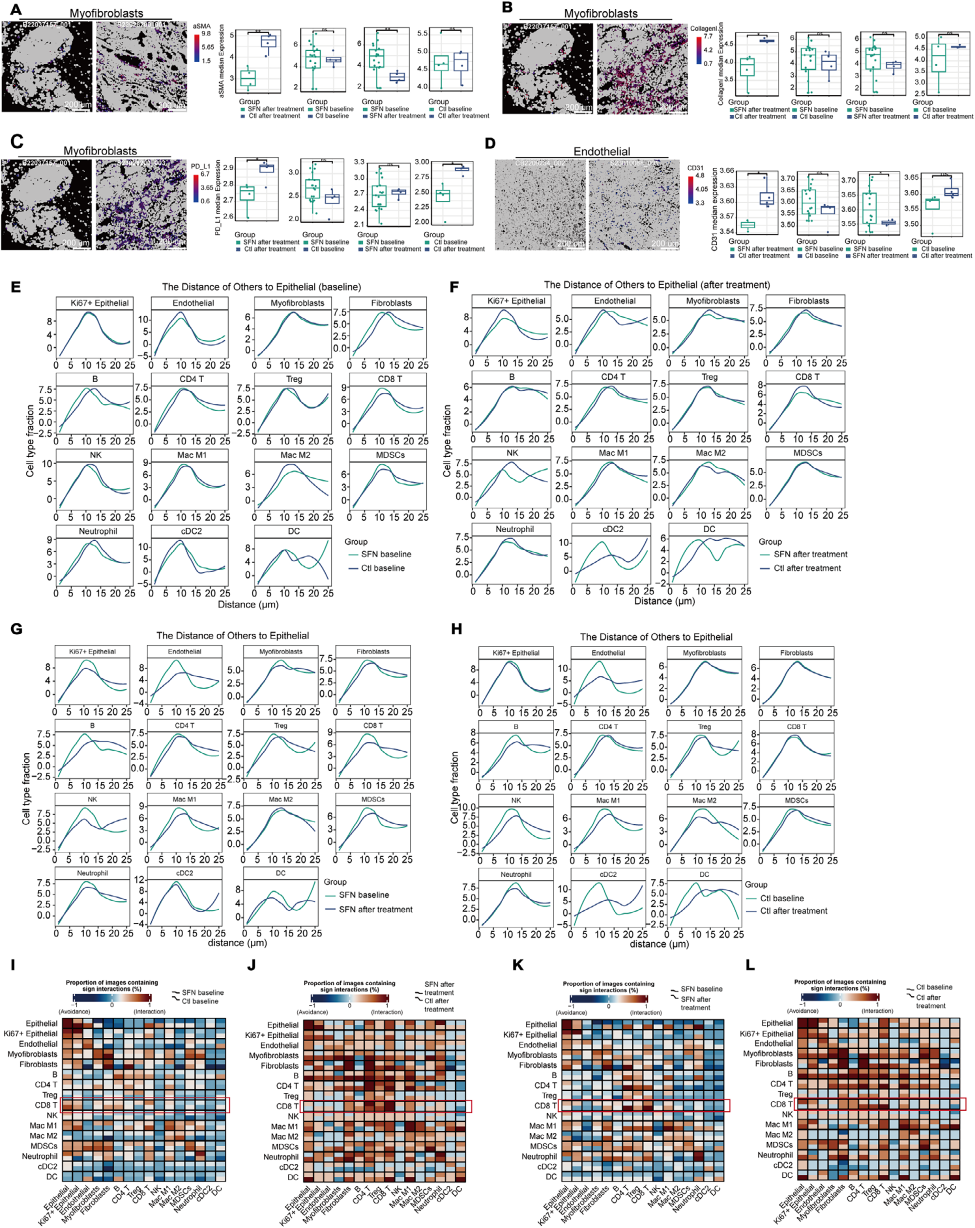
SFN treatment suppressed myofibroblast function and enhanced immune cell interplay in the tumor microenvironment. The levels of α‐SMA (A), collagen I (B) and PD‐L1 (C) on myofibroblasts were analyzed at the baseline, after treatment in control and experimental groups. (D) The level of CD31 on endothelia cells was analyzed at the baseline, after treatment in control and experimental groups. (E–H) Distances between other cell types to epithelial cells, with the cell type fraction calculated as the number of a specific cell type within 1 µm divided by the total number of that cell type in the slice. (I–L) Cell interactions at the baseline, after treatment in control and experimental groups (ns, nonsignificant difference; ^*^ *p* < 0.05; ^**^ *p* < 0.01; ^***^ *p* < 0.001).

Various studies have demonstrated the negative regulatory role of myofibroblasts in antitumor response, which depends on extracellular remodeling, blood vessel, and PD‐L1 expression to inhibit antitumor cell function [22, 23]. α‐SMA is an indicator for myofibroblast immune suppression [24]. SFN treatment significantly reduced α‐SMA levels based on the similar expression at baseline. In the experimental group, α‐SMA was significantly reduced after treatment compared with baseline; in the control group, there was no difference observed (Figure 5A). Myofibroblasts remodel the extracellular matrix through expressing matrix metallopeptidases MMPs or secreting collagen I [24]. We observed reduced collagen I levels after SFN treatment compared with control (Figure 5B). Following treatment, PD‐L1 on myofibroblasts was lower in the experimental group than the control group, and its levels were elevated in the control group after treatment (Figure 5C). SFN treatment reduced CD31 levels on endothelial compared with the control and experimental groups at baseline (Figure 5D). CD31 has been recognized as a blood vessel indicator [25]. These data suggested that SFN treatment may modulate myofibroblasts to regulate the extracellular matrix and blood vessels.

Extracellular matrix remodeling induces changes of immune cell interactions in the tumor microenvironment [26, 27]. The distances between most cells to epithelial did not exhibit difference at baseline in the control and experimental groups (Figure 5E). After treatment, CD8 T, NK, Type‐2 conventional dendritic cells (DC), and DC exhibited a closer distance in the experimental group compared with the control group (Figure 5F). SFN treatment shortened the distances of CD8^+^ T cells, M1, NK cells, neutrophils, and DC to epithelial (Figure 5G). In the control group, M2, Treg cells exhibited a closer distance after treatment (Figure 5H). Pearson's correlation analysis of the various cell types was performed to reveal the intercellular correlations at baseline and after treatment in the control and experimental groups. At baseline, the interactions between CD8^+^ T cells and epithelial, Ki67^+^ epithelial cells, fibroblasts, B cells, and DC were more active in the control group than the experimental group (Figure 5I). Following treatment, interactions between CD8^+^ T cells and tumor cells, CD4^+^ T cells, NK cells, and DC were strengthened, whereas those between MDSCs, B cells, and Treg cells were weakened (Figure 5J). SFN treatment enhanced the interactions between CD8^+^ T cells and CD4^+^ T cells, M1 and CD8^+^ T cells, whereas it reduced CD8^+^ T cell interplay with epithelial, Ki67^+^ epithelial cells, myofibroblasts, B cells, and MDSCs (Figure 5K). In the control group, the interplay between CD8 T cells and epithelial, Ki67^+^ epithelial cells, myofibroblasts, and DC was reduced, whereas the interactions between CD8^+^ T cells and endothelial, CD4^+^ T cells, Treg cells, M1, and neutrophils were enhanced after treatment compared with baseline (Figure 5L). These results indicated that SFN treatment suppressed the myofibroblast immunosuppression and enhanced immune cell interplay in the tumor microenvironment.

### 2.6 SFN Led to Spurious Progression of Lung Tumors

**FIGURE 6:**
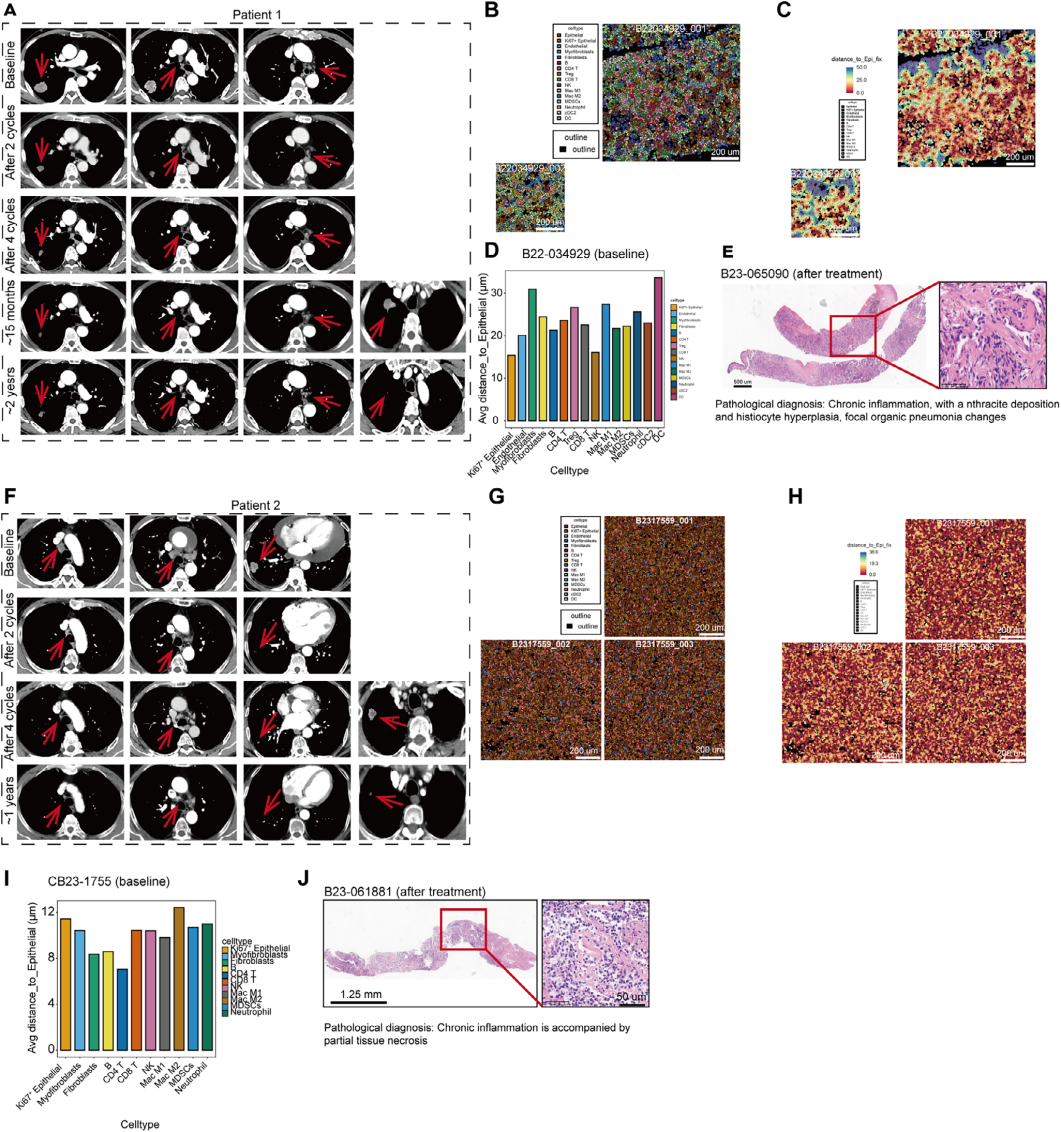
SFN leaded to spurious progression of lung tumors. (A) CT scans reveal a reduction in the tumor lesion of Patient 1 following SFN treatment. (B–D) IMC analysis of Patient 1 slide at the baseline. (E) H&E staining of Patient 1 slide at the baseline. (F) CT scans reveal a reduction in the tumor lesion of Patient 2 following SFN treatment. (G–I) IMC analysis of Patient 2 slide at the baseline. (J) H&E staining of Patient 2 slide at the baseline.

SFN treatment resulted in pseudoprogression during immunotherapy. Pseudoprogression was characterized by an initial increase in tumor size or the appearance of new lesions, which could be mistaken for disease progression. However, this phenomenon was often followed by a significant reduction in tumor burden without any change in treatment [28]. In the experimental group, two patients developed new lung lesions during treatment. One patient developed new lesions at 15 months of treatment (Figure 6A), while another patient developed new lesions after the fourth treatment cycle (Figure 6F). According to the IMC data, signals were detected in both slides at baseline, with higher proportions of Ki‐67^+^ epithelial, neutrophils, and MDSCs (Figure 6B–D, G–I). We conducted a tissue biopsy of the new lesions, and hematoxylin‐and‐eosin staining revealed inflammatory necrosis (Figure 6E,J). Upon continuing the original treatment regimen, the new lesions significantly shrank and nearly disappeared, maintaining this state for an extended period of time. This finding suggested that the combination of SFN with anti‐PD‐1 antibody and chemotherapy increased the abundance of immune cells in tissues and induced long survival.

## 3 Discussion

In the clinical setting, the efficacy of PD‐1 blockade in NSCLC remains far from satisfactory. This highlights the need for strategies that reshape the TME more sensitive to PD‐1 blockade [3]. Researchers have attempted to identify drugs that can synergize with PD‐1 blockade and revealed the importance of antitumor response in this process [29, 30]. Therefore, they focus on agents that may enhance antitumor response to improve PD‐1 blockade efficacy. Several strategies have been identified, including combination with chemotherapy, radiotherapy, targeted therapy, etc.

Numerous studies have explored candidates for combination treatment. Recently, the role of dietary factors in modulating immune response has been proposed. Among these, SFN received attention for its promising antitumor function [17]. SFN is a phytochemical derived from broccoli that has shown potential for chemoprevention and chemotherapy. It can prevent and control the occurrence and progression of various cancers, including prostate, lung, colorectal, and esophageal [31, 32, 33]. SFN can directly inhibit tumor migration and invasion [34], and play a role by regulating the immune function. In mice with metastatic melanoma, SFN significantly enhanced the activity of NK cells and the effect of antibody‐dependent cell‐mediated cytotoxicity, increased the levels of IL‐2 and IFN‐γ, and inhibited the spread of metastatic cancer [35, 36]. In this study, we extracted SFN from broccoli to investigate its function. The combination of anti‐PD‐1 antibody with chemotherapy serves as the first‐line treatment for NSCLC. To verify the antitumor pathway of SFN, we investigated the efficacy of SFN in combination with anti‐PD‐1 antibody and chemotherapy in subcutaneous tumor and tail vein metastatic tumor models. Consistent with previous evidence, we demonstrated that SFN could enhance CD8^+^ T cell infiltration and function, consequently synergizing with PD‐1 blockade in mouse models of LLC.

Previous studies [37, 38] showed that, compared with SFN and gefitinib alone, the combination of SFN and gefitinib significantly reduced the proliferation of gefitinib‐resistant PC9 lung cancer cells. Although studies have also investigated the combination of SFN with chemotherapy in other types of cancer [37], there is no research on the combination of SFN and immunotherapy. Our studies indicated that SFN synergistically enhanced the efficacy of chimeric antigen receptor‐T cells [20]. Based on the aforementioned study, we conducted a single‐center, observational, and prospective study to assess the safety and preliminary efficacy of SFN combined with anti‐PD‐1 antibody and chemotherapy. Patients in the triple‐agent group showed an improved clinical response (ORR and DCR) with longer PFS and OS than those in the control group. In addition, there were no new adverse events in the former group versus the latter group. These findings suggested that the combination of SFN with anti‐PD‐1 antibody and chemotherapy was safe and effective. We also investigated the synergistic mechanism of SFN and anti‐PD‐1 antibody.

Peripheral immune response is a positive indicator for PD‐1 blockade efficacy [39]. We observed innate immune response at baseline in the control and experimental groups; however, deep analysis revealed that the differences were mainly the protumor response. After treatment, the experimental group exhibited better antitumor response compared with the control group. Researchers attempted to maintain and enhance antitumor response to sensitize PD‐1 blockade. NK cells [40] and Type I interferon [41] play major roles in eliminating cancer cells. In this study, SFN treatment enhanced the Type I interferon signaling pathway and NK‐mediated cytotoxicity. The activation of caspases results in the cleavage and inactivation of key protein molecules, including the DNA repair enzyme poly (ADP‐ribose) polymerase (PARP), and facilitates the release of mitochondrial apoptosis‐inducing factors (AIF). Once released, AIF and transcription factors such as NF‐kB are upregulated, all of which play roles in the induction of cell survival genes. SFN promotes apoptosis by regulating the functions of these factors [42]. Moreover, SFN treatment elevated other antitumor pathways, including regulation of extrinsic apoptotic signaling pathway and leukocyte transendothelial migration. The control group exhibited reduced Type I interferon signaling pathway after treatment compared with baseline. After SFN treatment, patients exhibited IFN‐γ‐mediated signaling pathway and antigen processing and presentation of exogenous peptide antigen, which contributed to antitumor response [43, 44]. These data were obtained from the RNA‐seq of peripheral blood samples extracted from patients with NSCLC. MDSCs are a negative indicator of tumor progression and PD‐1 blockade [45, 46]. Previously, we reported that SFN could activate CD8^+^ T cells through inducing MDSC apoptosis [21]. Consistently, based on flow data, we observed that SFN treatment elevated the CD8^+^ T cell proportions and functions, while reducing Treg cells and MDSCs. This suggests that SFN could enhance the antitumor peripheral immune response.

Analysis of the tumor microenvironment demonstrated that SFN treatment elevated the CD8^+^ T‐cell infiltration and function compared with control. Ki67 is an indicator for proliferating T and NK cells. We observed higher Ki67 levels on CD4^+^, CD8^+^ T, and NK cells after treatment in the experimental group. Granzyme B plays a major role in antitumor cells; we also found higher expression of granzyme B on CD4^+^, CD8^+^ T, and NK cells after treatment in the experimental group. Reduced PD‐L1 (negative indicator of antitumor response) was observed on M2, MDSCs, and myofibroblasts after treatment in the experimental group. Tumor progression and clinical failure are often accompanied with more blood vessels [47]. SFN treatment reduced CD31 on endothelial (indicator of blood vessel [48]) compared with control. More immune cells interactions were observed after SFN treatment versus control. Thus, SFN treatment could enhance antitumor response while reducing immunosuppressive cells’ function and suppressing blood vessels.

During anti‐PD‐1 antibody treatment, imaging may show a temporary increase in tumor volume or the appearance of new lesions. This indicates a local response due to immune cell infiltration, inflammation, or necrosis rather than actual tumor progression [49]. Pseudoprogression occurred within the first 2 months after immunotherapy, and the incidence rate was approximately 3%–8% [50]. In this study, two patients developed new lesions in the lungs, which were histologically confirmed as inflammatory necrosis. These lesions occurred after 15 months of treatment and after the fourth treatment cycle, respectively. Patients experiencing pseudoprogression may exhibit improved long‐term survival rates; therefore, they should be evaluated to prevent premature treatment cessation [51]. The onset of pseudoprogression during SFN combination therapy was delayed compared with previous studies. This could account for the better PFS and OS by those treated with SFN combination therapy.

Currently, there are no other data available from prospective studies assessing SFN as a PD‐1 blockade that achieved the best tumor control, suggesting that SFN as a neoadjuvant could sensitize PD‐1 blockade in NSCLC. Nonetheless, this study had limitations. First, the patients received only four cycles of SFN treatment. For cases with long PFS, it may be necessary to explore the relevant mechanisms to determine the role of SFN. Second, previous studies reported pseudoprogression after PD‐1 blockade monotherapy; in this study, pseudoprogression occurred in the experimental group. It is unknown whether this is related to SFN treatment. The encouraging safety and efficacy profile of SFN may facilitate immunotherapy options in NSCLC in which suboptimal exposures to PD‐1 blockade have prevented clinical development. Therefore, SFN may offer clinical options to patients with NSCLC who have progressed on prior therapy with PD‐1/PD‐L1 inhibitors.

In summary, this study provides preliminary evidence that SFN, used as a neoadjuvant, can enhance the sensitivity of NSCLC to PD‐1 blockade, achieving favorable tumor control outcomes. The observed safety profile further supports its potential application in clinical settings. However, the limitations identified, such as the fixed duration of SFN treatment and the uncertainty surrounding pseudoprogression in the experimental group, highlight the need of future investigations. Such efforts may pave the way for more effective and personalized immunotherapeutic strategies in the management of NSCLC.

## 4 Materials and Methods

### 4.1 Study Design and Assessment

This study enrolled 80 patients pathologically diagnosed with NSCLC between June 2021 and November 2023. The main inclusion criteria for all patients included age 18–70 years, an Eastern Cooperative Oncology Group (ECOG) performance status score of 0–1, adequate organ function, and at least one measurable lesion according to the Response Evaluation Criteria in Solid Tumors (RECIST) version 1.1. The main exclusion criteria included: history of bone marrow or solid organ transplantation; autoimmune diseases that are active or have recently required systemic immunosuppressive therapy; history of severe hypersensitivity to monoclonal antibodies, gemcitabine, pemetrexed, paclitaxel, and platinum; and prior treatment with monoclonal antibodies targeting PD‐1/PD‐L1/CTLA‐4.

### 4.2 SFN

SFN was extracted from broccoli seeds by the plant synthase method. Broccoli seeds were obtained from the Chinese Academy of Agricultural Sciences. SFN‐free seed extracts were used as negative control, obtained after the removal of key active enzymes. The results of liquid chromatography indicated that the mass percentage of SFN was 3.0678%, and the negative control was 0.75 mg/mL.

### 4.3 Mouse Model

To construct a subcutaneous xenograft mouse model, mice were divided into different groups (*n* = 5 per group) randomly: control group (distilled water); negative control group (SFN‐free); experimental group (SFN 90 µmol/kg gavage per day; Pu'er Qiyun Biotechnology Co. Ltd.); DTX group (10 mg/kg intraperitoneally once per week; Selleck Chemicals); anti‐PD‐1 antibody group (200 µg/mouse intraperitoneally once every 3 days; Bio X Cell); SFN+anti‐PD‐1 group; SFN+DTX group; DTX+anti‐PD‐1 antibody group; and SFN+DTX+anti‐PD‐1 antibody group. Each group received hypodermic injections of LLC cells (1 × 10^6^ cells in 100 µL of phosphate‐buffered saline [PBS]). Tumor growth was monitored by measuring the tumor volume ([length × width^2^]/2) with calipers every 3 days, and body weight was recorded once daily to assess the general health status of the mice. All mice were euthanized when the tumor volume reached the ethical endpoint or at the end of the experimental period, and tumors were harvested for further analysis.

We injected 1 × 10^6^ luciferase‐labeled fluorescent protein‐LLC cells into the tail vein. For in vivo determination of the tumor burden, mice were anesthetized and then injected with 75 mg/kg D‐luciferin (100 mL of 30 mg/mL in PBS) 10 min prior to imaging intraperitoneally. IVIS Spectrum Imaging System (PerkinElmer) was used to image tumor growth. And then, Living Image software program (PerkinElmer) was used to calculate. Once the bioluminescence imaging signal reaching 1e5–1e7, the mice were randomly assigned to control group (distilled water); negative control group (SFN‐free); experimental group (SFN 90 µmol/kg gavage per day; Pu'er Qiyun Biotechnology Co. Ltd.); DTX group (10 mg/kg intraperitoneally once per week; Selleck Chemicals); anti‐PD‐1 antibody group (200 µg/mouse intraperitoneally once every 3 days; Bio X Cell); SFN+anti‐PD‐1 group; SFN+DTX group; DTX+anti‐PD‐1 antibody group; and SFN+DTX+anti‐PD‐1 antibody group.

### 4.4 Obtaining Single Cell From Tumor Tissue

The mice were euthanized by cervical dislocation, and the tumor was extracted; subsequently, the hair attached to the tumor was removed, followed by washing with PBS. The 5 mm of tissues were cut without blocking the straw; a digestive solution containing three enzymes (collagenase, hyaluronidase, and deoxyribonuclease I) (500 µL) was added, and the tumor was digested at 37°C for 30 min. Digestion was blocked with RPMI 1640 complete culture medium. After filtering with a sieve to remove undigested tissue blocks, the cell suspension containing the digestive solution and culture medium was collected. Following centrifugation at 1500 rpm for 5 min, the supernatant was removed.

### 4.5 Antibodies and Flow Cytometry

CytoFLEX flow cytometer (Beckman Coulter, Brea, CA, USA) was used for flow cytometry. The cells were stained with fluorescence‐conjugated antibodies of surface marker at 4°C for 30 min in the dark. Then, the cells were fixed and permeabilized and followed by staining with antibodies against intracellular cytokine. All antibodies were purchased from BioLegend (San Diego, CA, USA): fluorescein isothiocyanate (FITC) anti‐mouse CD45 (30‐F11); BV510 anti‐mouse CD3 (17A2); PerCP/Cyanine5.5 anti‐mouse CD4 (GK1.5); BV 421 anti‐mouse IFN‐γ antibody (XMG1.2); BV 785 anti‐mouse TNF‐α; (MP6‐XT22) AF 700 anti‐mouse CD8a (53‐6.7); and BV 785 anti‐mouse CD279 (29F.1A12).

We collected peripheral blood samples from patients at baseline and after treatment for flow cytometry. Single‐cell suspensions were stained with the following antibodies: ECD‐A cell viability; KO525 anti‐human CD45; APC‐A700 anti‐human CD8; FITC anti‐human CD3; PB450 anti‐human CD69; PC7 anti‐human CTLA‐4; and phycoerythrin (PE) anti‐human PD‐1 (BioLegend). For intracellular staining, cells were stimulated for 4–6 h at 37°C with phorbol myristate acetate (50 ng/mL) and ionomycin (1 µg/mL). After fixation and permeabilization, the cells were stained with KO525 anti‐human CD45, APC‐A700 anti‐human CD8, FITC anti‐human CD3, and APC anti‐human IFN‐γ (BioLegend). For MDSCs, cells were stained with KO525 cell viability, APC anti‐human HLA‐DR, FITC anti‐human CD11b, APC‐A700 anti‐human CD14, PC5.5 anti‐human IL‐8, violet610 anti‐human CD73, and PB450 anti‐human PD‐L1 (BioLegend). For Treg cells, cells were stained with KO525 anti‐human CD45, PC5.5 anti‐human CD4, APC anti‐human CD25, and PE anti‐human FOXP3 (BioLegend). Flow cytometry analysis was performed using a CytExpert for DxFLEX (Beckman Coulter).

### 4.6 RNA‐seq Analysis

We collected blood samples from patients at baseline and after SFN treatment. Subsequently, PBMCs were obtained and stored in TRIzol (catalog number: 9109; Takara, Japan). The samples were sent to Wuhan Kangce Company for RNA‐seq (contract number: KC2023‐H0336). Data were analyzed using Gene Ontology (GO)/Kyoto Encyclopedia of Genes and Genomes (KEGG)/Gene Set Enrichment Analysis (GSEA) based on the differentially expressed genes.

### 4.7 IMC Analysis

We intended to conduct a second tissue biopsy following four cycles of treatment or upon disease progression. However, not all patients agreed to a second biopsy during the treatment period, leading to a scarcity of samples. Formalin‐fixed paraffin‐embedded NSCLC sections (5–8 mm) were collected from the Pathology Department of The First Affiliated Hospital of Zhengzhou University. The slides were sent to Nanjing Aoyin Biotechnology Co. Ltd. (Jiangsu, China) for IMC. The slides were stained with a cocktail of 32 antibodies. As previously reported (single‐cell analysis of the human pancreas in Type 2 diabetes using multi‐spectral IMC), after acquiring the IMC data (MCD and text file format) from the Hyperion Imaging System, a Fluidigm MCD viewer (v1.0.560.2) was used to visualize the marker expression of each region of interest on the tissue sections. Images were exported as 16‐bit.ome.tiff files from MCD Viewer and imported into Cell Profiler v4.1.2. Followed by individual nuclei, define cell borders, and generate cell masks identification. HistoCAT v1.7.6 software was used to extract single cell features from these images. Harmony v 0.1.0 was used to eliminate batch effects. ComplexHeatmap v2.10.0 was used to generate heatmap to demonstrate the median z score (0–1) of marker expression in cells from each cluster. For dimensionality reduction, t‐stochastic neighbor embedding (t‐SNE) visualization was used to determine the phenotypic diversity of the cell populations. Cytomapper v1.6.0 was utilized to support the handling and visualization of multiple multichannel images and segmentation masks. In addition, spatial analysis and neighborhood analysis were performed with imcRtools v1.1.7.

Samples from 13 patients were obtained and 37 ROI were examined; however, 6 ROI were excluded for the large area nonspecific staining. To calculate the protein expression, the median value of the selected protein expressed on a certain type of cell in each sample should be obtained, then compared the difference between groups. Comparison of cell type scores within 25 µm: First, calculate the total number of cells of a certain type within 25 µm. Then, calculate the number of cells of that type within each 1‐µm interval. Cell type score: the proportion of cells of that type within this micrometer interval to the total number of cells of that type within the 25‐µm interval (for example: cell type fraction of CD8 T in the 0–1‐μ range = number of CD8 T in the 0–1‐μ range). Based on the comparison of cell proportions between groups at the sample level, a vertical axis title (Cells Proportion) is added here, with a total cell proportion of 1. Calculate the proportion of each cell type in the sample, obtain the proportion data, and then compare the cell proportion between groups based on the sample level.

### 4.8 Statistical Analysis

All data are expressed as the mean ± SEM. The differences between groups were analyzed by paired or unpaired Student's *t*‐test. The safety analysis data were analyzed using a paired *t*‐test within the group and a non‐paired *t*‐test between groups. Statistical analysis was performed by GraphPad Prism V.9 (San Diego, CA, USA). The *p*‐values < 0.05 indicate statistically significant differences.

## Author Contributions

J. Li and J. Liu conceptualized the study. M.Z. and Z.W. developed the methodology. M.Y., Z.F., C.G., and T.Z. curated and analyzed the data. S.L. and J.H. performed the statistical analyses. D.Y. and Y. Li provided the technical and material support. S.Y., Q.G., Y. Liu, and L.W. provided the clinical samples. Y.Z. and L.W. supervised the study. J. Li drafted the manuscript. Y.Z. is responsible for the overall content as the guarantor. All the authors revised and approved the final manuscript.

## Funding

This study was approved by the National Natural Science Foundation of China Joint Fund Key Support Project (U24A20734), the Key Special Project for International Science and Technology Innovation Cooperation between Governments under the National Key R&D Program (2022YFE0141000), and the National Natural Science Foundation of China—General Project (82272873).

## Ethics Statement

All animal procedures were conducted in accordance with the Guide for the Care and Use of Laboratory Animals and were approved by the Institutional Animal Care and Use committee of The First Affiliated Hospital of Zhengzhou University (2021071001). This clinical trial was approved by the Clinical Research Ethics Committee of The First Affiliated Hospital of Zhengzhou University (KY‐2021‐0266).

## Conflicts of Interest

The authors declare no conflicts of interest.

## Supporting information




**Figure S1**: Trial profile and safety analysis.
**Figure S2**: SFN treatment enhance the anti‐tumor response in peripheral.
**Figure S3**: IMC analysis of tumor microenvironment.
**Figure S4**: The cell proportions difference was analyzed.
**Table S1**: Baseline characteristics of NSCLC patients with sequential and simultaneous combination before and after matching.
**Table S2**: Treatment‐related adverse events and serious treatment‐related adverse events in each group.

## Data Availability

The authors confirm that the data supporting the findings of this study are available within NGDC (OMIX014594, https://ngdc.cncb.ac.cn/omix/submitList).

## References

[1] J. C. Soria , Y. Ohe , J. Vansteenkiste , et al., “Osimertinib in Untreated EGFR‐Mutated Advanced Non‐Small‐Cell Lung Cancer,” New England Journal of Medicine 378 (2018): 113–125. 29151359 10.1056/NEJMoa1713137

[2] G. V. Scagliotti , P. Parikh , J. von Pawel , et al., “Phase III Study Comparing Cisplatin Plus Gemcitabine With Cisplatin Plus Pemetrexed in Chemotherapy‐Naive Patients With Advanced‐Stage Non‐Small‐Cell Lung Cancer,” Journal of Clinical Oncology 26 (2008): 3543–3551. 18506025 10.1200/JCO.2007.15.0375

[3] L. Gandhi , D. Rodríguez‐Abreu , S. Gadgeel , et al., “Pembrolizumab Plus Chemotherapy in Metastatic Non‐Small‐Cell Lung Cancer,” New England Journal of Medicine 378 (2018): 2078–2092. 29658856 10.1056/NEJMoa1801005

[4] M. A. Socinski , M. Nishio , R. M. Jotte , et al., “IMpower150 Final Overall Survival Analyses for Atezolizumab Plus Bevacizumab and Chemotherapy in First‐Line Metastatic Nonsquamous NSCLC,” Journal of Thoracic Oncology 16 (2021): 1909–1924. 34311108 10.1016/j.jtho.2021.07.009

[5] M. A. Socinski , R. M. Jotte , F. Cappuzzo , et al., “Atezolizumab for First‐Line Treatment of Metastatic Nonsquamous NSCLC,” New England Journal of Medicine 378 (2018): 2288–2301. 29863955 10.1056/NEJMoa1716948

[6] H. West , M. McCleod , M. Hussein , et al., “Atezolizumab in Combination With Carboplatin Plus Nab‐Paclitaxel Chemotherapy Compared With Chemotherapy Alone as First‐Line Treatment for Metastatic Non‐Squamous Non‐Small‐Cell Lung Cancer (IMpower130): A Multicentre, Randomised, Open‐Label, Phase 3 Trial,” Lancet Oncology 20 (2019): 924–937. 31122901 10.1016/S1470-2045(19)30167-6

[7] C. Zhou , G. Chen , Y. Huang , et al., “Camrelizumab Plus Carboplatin and Pemetrexed Versus Chemotherapy Alone in Chemotherapy‐Naive Patients With Advanced Non‐Squamous Non‐Small‐Cell Lung Cancer (CameL): A Randomised, Open‐Label, Multicentre, Phase 3 Trial,” Lancet Respiratory Medicine 9 (2021): 305–314. 33347829 10.1016/S2213-2600(20)30365-9

[8] M. J. Grant , R. S. Herbst , and S. B. Goldberg , “Selecting the Optimal Immunotherapy Regimen in Driver‐Negative Metastatic NSCLC,” Nature Reviews Clinical Oncology 18 (2021): 625–644. 10.1038/s41571-021-00520-1 34168333

[9] E. L. Leung , R. Z. Li , X. X. Fan , et al., “Longitudinal High‐Dimensional Analysis Identifies Immune Features Associating With Response to Anti‐PD‐1 Immunotherapy,” Nature Communications 14 (2023): 5115. 10.1038/s41467-023-40631-0 PMC10444872 37607911

[10] Y. Liu , Y. Ping , L. Zhang , et al., “Changes in L‐Phenylalanine Concentration Reflect and Predict Response to Anti‐PD‐1 Treatment Combined With Chemotherapy in Patients With Non‐Small Cell Lung Cancer,” MedComm 6 (2025): e70100. 39968502 10.1002/mco2.70100 PMC11832432

[11] J. Huang , D. Liu , Y. Wang , et al., “Ginseng Polysaccharides Alter the Gut Microbiota and Kynurenine/Tryptophan Ratio, Potentiating the Antitumour Effect of Antiprogrammed Cell Death 1/Programmed Cell Death Ligand 1 (Anti‐PD‐1/PD‐L1) Immunotherapy,” Gut 71 (2022): 734–745. 34006584 10.1136/gutjnl-2020-321031 PMC8921579

[12] P. Yu , H. Wei , K. Li , et al., “The Traditional Chinese Medicine Monomer Ailanthone Improves the Therapeutic Efficacy of Anti‐PD‐L1 in Melanoma Cells by Targeting c‐Jun,” Journal of Experimental & Clinical Cancer Research 41 (2022): 346. 36522774 10.1186/s13046-022-02559-z PMC9753288

[13] S. Zwirner , A. A. Abu Rmilah , S. Klotz , et al., “First‐in‐Class MKK4 Inhibitors Enhance Liver Regeneration and Prevent Liver Failure,” Cell 187 (2024): 1666–1684.e1626. 38490194 10.1016/j.cell.2024.02.023 PMC11011246

[14] Y. Xu , X. Han , Y. Li , et al., “Sulforaphane Mediates Glutathione Depletion via Polymeric Nanoparticles to Restore Cisplatin Chemosensitivity,” ACS Nano 13 (2019): 13445–13455. 31670945 10.1021/acsnano.9b07032

[15] Y. Ge , Z. Ge , F. Tian , et al., “Sulforaphane Potentiates the Efficacy of Chemoradiotherapy in Glioblastoma by Selectively Targeting Thioredoxin Reductase 1,” Cancer Letters 611 (2024): 217429. 39725145 10.1016/j.canlet.2024.217429

[16] Z. Lu , Y. Ren , L. Yang , et al., “Inhibiting Autophagy Enhances Sulforaphane‐Induced Apoptosis via Targeting NRF2 in Esophageal Squamous Cell Carcinoma,” Acta Pharmaceutica Sinica B 11 (2021): 1246–1260. 34094831 10.1016/j.apsb.2020.12.009 PMC8148075

[17] Q. Zou , X. Zhou , J. Lai , et al., “Targeting p62 by Sulforaphane Promotes Autolysosomal Degradation of SLC7A11, Inducing Ferroptosis for Osteosarcoma Treatment,” Redox Biology 79 (2025): 103460. 39657365 10.1016/j.redox.2024.103460 PMC11681892

[18] Q. Shi , Y. Liu , W. Yang , Y. Li , C. Wang , and K. Gao , “The Covalent Modification of STAT1 Cysteines by Sulforaphane Promotes Antitumor Immunity via Blocking IFN‐γ‐Induced PD‐L1 Expression,” Redox Biology 81 (2025): 103543. 39961271 10.1016/j.redox.2025.103543 PMC11875811

[19] I. Xagoraris , Y. Yang , E. Bougka , et al., “Sulforaphane Promotes Natural Killer Cell‐Mediated Anti‐Tumor Immune Responses Partially via cGAS‐STING Pathway in Classical Hodgkin lymphoma,” Leukemia 39 (2025): 1787–1790. 40295827 10.1038/s41375-025-02627-1 PMC12208876

[20] C. Shen , Z. Zhang , Y. Tian , et al., “Sulforaphane Enhances the Antitumor Response of Chimeric Antigen Receptor T Cells by Regulating PD‐1/PD‐L1 Pathway,” BMC Medicine 19 (2021): 283. 34819055 10.1186/s12916-021-02161-8 PMC8614004

[21] J. Liu , H. Chen , C. Guo , et al., “Sulforaphane Activates CD8(+) T Cells Antitumor Response Through IL‐12RB2/MMP3/FasL‐Induced MDSCs Apoptosis,” Journal for ImmunoTherapy of Cancer 12 (2024): e007983. 38296593 10.1136/jitc-2023-007983 PMC10831471

[22] A. T. Krishnamurty , J. A. Shyer , M. Thai , et al., “LRRC15(+) Myofibroblasts Dictate the Stromal Setpoint to Suppress Tumour Immunity,” Nature 611 (2022): 148–154. 36171287 10.1038/s41586-022-05272-1 PMC9630141

[23] L. Sun , Y. Wang , X. Wang , et al., “PD‐L1 Promotes Myofibroblastic Activation of Hepatic Stellate Cells by Distinct Mechanisms Selective for TGF‐β Receptor I Versus II,” Cell Reports 38 (2022): 110349. 35139382 10.1016/j.celrep.2022.110349 PMC8903892

[24] Y. Chen , J. Kim , S. Yang , et al., “Type I Collagen Deletion in αSMA(+) Myofibroblasts Augments Immune Suppression and Accelerates Progression of Pancreatic Cancer,” Cancer Cell 39 (2021): 548–565.e546. 33667385 10.1016/j.ccell.2021.02.007 PMC8423173

[25] T. Fu , D. P. Sullivan , A. M. Gonzalez , et al., “Mechanotransduction via Endothelial Adhesion Molecule CD31 Initiates Transmigration and Reveals a Role for VEGFR2 in Diapedesis,” Immunity 56 (2023): 2311–2324.e2316. 37643615 10.1016/j.immuni.2023.08.001 PMC11670454

[26] H. Zhang , C. K. Tsui , G. Garcia , et al., “The Extracellular Matrix Integrates Mitochondrial Homeostasis,” Cell 187 (2024): 4289–4304.e4226. 38942015 10.1016/j.cell.2024.05.057 PMC12352124

[27] T. E. Sutherland , D. P. Dyer , and J. E. Allen , “The Extracellular Matrix and the Immune System: A Mutually Dependent Relationship,” Science 379 (2023): eabp8964. 36795835 10.1126/science.abp8964

[28] Q. Wang , J. Gao , and X. Wu , “Pseudoprogression and Hyperprogression After Checkpoint Blockade,” International Immunopharmacology 58 (2018): 125–135. 29579717 10.1016/j.intimp.2018.03.018

[29] N. Liu , L. Chen , M. Yan , et al., “ *Eubacterium rectale* Improves the Efficacy of Anti‐PD1 Immunotherapy in Melanoma via L‐Serine‐Mediated NK Cell Activation,” Research 6 (2023): 0127. 37223471 10.34133/research.0127 PMC10202379

[30] Y. Ping , J. Shan , H. Qin , et al., “PD‐1 Signaling Limits Expression of Phospholipid Phosphatase 1 and Promotes Intratumoral CD8(+) T Cell Ferroptosis,” Immunity 57 (2024): 2122–2139.e2129. 39208806 10.1016/j.immuni.2024.08.003

[31] M. Russo , C. Spagnuolo , G. L. Russo , et al., “Nrf2 Targeting by Sulforaphane: A Potential Therapy for Cancer Treatment,” Critical Reviews in Food Science and Nutrition 58 (2018): 1391–1405. 28001083 10.1080/10408398.2016.1259983

[32] X. Haristoy , K. Angioi‐Duprez , A. Duprez , and A. Lozniewski , “Efficacy of Sulforaphane in Eradicating *Helicobacter pylori* in Human Gastric Xenografts Implanted in Nude Mice,” Antimicrobial Agents and Chemotherapy 47 (2003): 3982–3984. 14638516 10.1128/AAC.47.12.3982-3984.2003 PMC296232

[33] X. Liu and K. Lv , “Cruciferous Vegetables Intake Is Inversely Associated With Risk of Breast Cancer: A Meta‐Analysis,” Breast 22 (2013): 309–313. 22877795 10.1016/j.breast.2012.07.013

[34] Y. Li , T. Zhang , H. Korkaya , et al., “Sulforaphane, a Dietary Component of Broccoli/Broccoli Sprouts, Inhibits Breast Cancer Stem Cells,” Clinical Cancer Research 16 (2010): 2580–2590. 20388854 10.1158/1078-0432.CCR-09-2937 PMC2862133

[35] S. V. Singh , R. Warin , D. Xiao , et al., “Sulforaphane Inhibits Prostate Carcinogenesis and Pulmonary Metastasis in TRAMP Mice in Association With Increased Cytotoxicity of Natural Killer Cells,” Cancer Research 69 (2009): 2117–2125. 19223537 10.1158/0008-5472.CAN-08-3502 PMC2683380

[36] P. Thejass and G. Kuttan , “Modulation of Cell‐Mediated Immune Response in B16F‐10 Melanoma‐Induced Metastatic Tumor‐Bearing C57BL/6 Mice by Sulforaphane,” Immunopharmacology and Immunotoxicology 29 (2007): 173–186. 17849266 10.1080/08923970701511728

[37] F. Wang , W. Wang , J. Li , J. Zhang , X. Wang , and M. Wang , “Sulforaphane Reverses Gefitinib Tolerance in Human Lung Cancer Cells via Modulation of Sonic Hedgehog Signaling,” Oncology Letters 15 (2018): 109–114. 29285189 10.3892/ol.2017.7293 PMC5738694

[38] F. Wang , Y. Sun , X. Huang , et al., “Sulforaphane Inhibits Self‐Renewal of Lung Cancer Stem Cells Through the Modulation of Sonic Hedgehog Signaling Pathway and Polyhomeotic Homolog 3,” AMB Express 11 (2021): 121. 34424425 10.1186/s13568-021-01281-x PMC8382806

[39] D. Dyikanov , A. Zaitsev , T. Vasileva , et al., “Comprehensive Peripheral Blood Immunoprofiling Reveals Five Immunotypes With Immunotherapy Response Characteristics in Patients With Cancer,” Cancer Cell 42 (2024): 759–779.e712. 38744245 10.1016/j.ccell.2024.04.008

[40] R. B. Delconte , M. Owyong , E. K. Santosa , et al., “Fasting Reshapes Tissue‐Specific Niches to Improve NK Cell‐Mediated Anti‐Tumor Immunity,” Immunity 57 (2024): 1923–1938.e1927. 38878769 10.1016/j.immuni.2024.05.021 PMC11684419

[41] J. D. Klement , P. S. Redd , C. Lu , et al., “Tumor PD‐L1 Engages Myeloid PD‐1 to Suppress Type I Interferon to Impair Cytotoxic T Lymphocyte Recruitment,” Cancer Cell 41 (2023): 620–636.e629. 36917954 10.1016/j.ccell.2023.02.005 PMC10150625

[42] Y. Zhou , G. Yang , H. Tian , et al., “Sulforaphane Metabolites Cause Apoptosis via Microtubule Disruption in Cancer,” Endocrine‐Related Cancer 25 (2018): 255–268. 29431641 10.1530/ERC-17-0483

[43] Y. Han , C. Zou , T. Liu , W. Cheng , P. Cheng , and A. Wu , “Inhibiting Interferon‐γ Induced Cancer Intrinsic TNFRSF14 Elevation Restrains the Malignant Progression of Glioblastoma,” Journal of Experimental & Clinical Cancer Research 43 (2024): 212. 39085878 10.1186/s13046-024-03131-7 PMC11289992

[44] S. Jhunjhunwala , C. Hammer , and L. Delamarre , “Antigen Presentation in Cancer: Insights Into Tumour Immunogenicity and Immune Evasion,” Nature Reviews Cancer 21 (2021): 298–312. 33750922 10.1038/s41568-021-00339-z

[45] H. Zhao , D. Teng , L. Yang , et al., “Myeloid‐Derived Itaconate Suppresses Cytotoxic CD8(+) T Cells and Promotes Tumour Growth,” Nature Metabolism 4 (2022): 1660–1673. 10.1038/s42255-022-00676-9 PMC10593361 36376563

[46] Y. Liu , C. C. Wong , Y. Ding , et al., “ *Peptostreptococcus Anaerobius* Mediates Anti‐PD1 Therapy Resistance and Exacerbates Colorectal Cancer via Myeloid‐Derived Suppressor Cells in Mice,” Nature Microbiology 9 (2024): 1467–1482. 10.1038/s41564-024-01695-w PMC11153135 38750176

[47] A. U. Kabir , C. Zeng , M. Subramanian , et al., “ZBTB46 Coordinates Angiogenesis and Immunity to Control Tumor Outcome,” Nature Immunology 25 (2024): 1546–1554. 39134750 10.1038/s41590-024-01936-4 PMC13355241

[48] E. E. Crouch , A. Bhaduri , M. G. Andrews , et al., “Ensembles of Endothelial and Mural Cells Promote Angiogenesis in Prenatal Human Brain,” Cell 185 (2022): 3753–3769.e3718. 36179668 10.1016/j.cell.2022.09.004 PMC9550196

[49] W. Jia , Q. Gao , A. Han , H. Zhu , and J. Yu , “The Potential Mechanism, Recognition and Clinical Significance of Tumor Pseudoprogression After Immunotherapy,” Cancer Biology & Medicine 16 (2019): 655–670. 31908886 10.20892/j.issn.2095-3941.2019.0144 PMC6936240

[50] D. Fujimoto , H. Yoshioka , Y. Kataoka , et al., “Pseudoprogression in Previously Treated Patients with Non‐Small Cell Lung Cancer Who Received Nivolumab Monotherapy,” Journal of Thoracic Oncology 14 (2019): 468–474. 30468872 10.1016/j.jtho.2018.10.167

[51] J. H. Lee , G. V. Long , A. M. Menzies , et al., “Association Between Circulating Tumor DNA and Pseudoprogression in Patients With Metastatic Melanoma Treated With Anti‐Programmed Cell Death 1 Antibodies,” JAMA Oncology 4 (2018): 717–721. 29423503 10.1001/jamaoncol.2017.5332 PMC5885201

