# Supplementary material for: Sulforaphane Synergizes With PD‐1 Blockade Through Activating CD8+ T Cells in Non–Small Cell Lung Cancer: Preclinical and Clinical Investigations
Source: MedComm (2020). 2026 Mar 24;7(4):e70688. doi: 10.1002/mco2.70688 (PMC13042748; doi:10.1002/mco2.70688)
Supplement: Supplementary file 1 — Figure S1: Trial profile and safety analysis. Figure S2: SFN treatment enhance the anti‐tumor response in peripheral. Figure S3: IMC analysis of tumor microenvironment. Figure S4: The cell proportions difference was analyzed. Table S1: Baseline characteristics of NSCLC patients with sequential and simultaneous combination before and after matching. Table S2: Treatment‐related adverse events and serious treatment‐related adverse events in each group. [file MCO2-7-e70688-s001.doc]

Supplementary Materials for

**Sulforaphane synergizes with PD-1 blockade through activating CD8+ T cells in non-small cell lung cancer: pre-clinical and clinical investigations**

Jieyao Li^#1,2^, Jinyan Liu^#1^, Zheng Wang^#1,2^, Ming Zhao^#1^, Mingming You^1^, Ziyi Fu^1^, Caijuan Guo^1^, Tengyue Zhang^1^, Shasha Liu^1^, Dongli Yue^2^, Shuangning Yang^1^, Yixin Li^2^, Qun Gao^2^, Yanfen Liu^1^, Jianmin Huang^1^, Liping Wang*^2^, Yi Zhang*^1,3,4,5,6^

Correspondence to: [yizhang@zzu.edu.cn](mailto:yizhang@zzu.edu.cn) and wlp@zzu.edu.cn

**This PDF file includes:**

Figures S1 to S4

Tables S1 to S2

Materials and methods

**
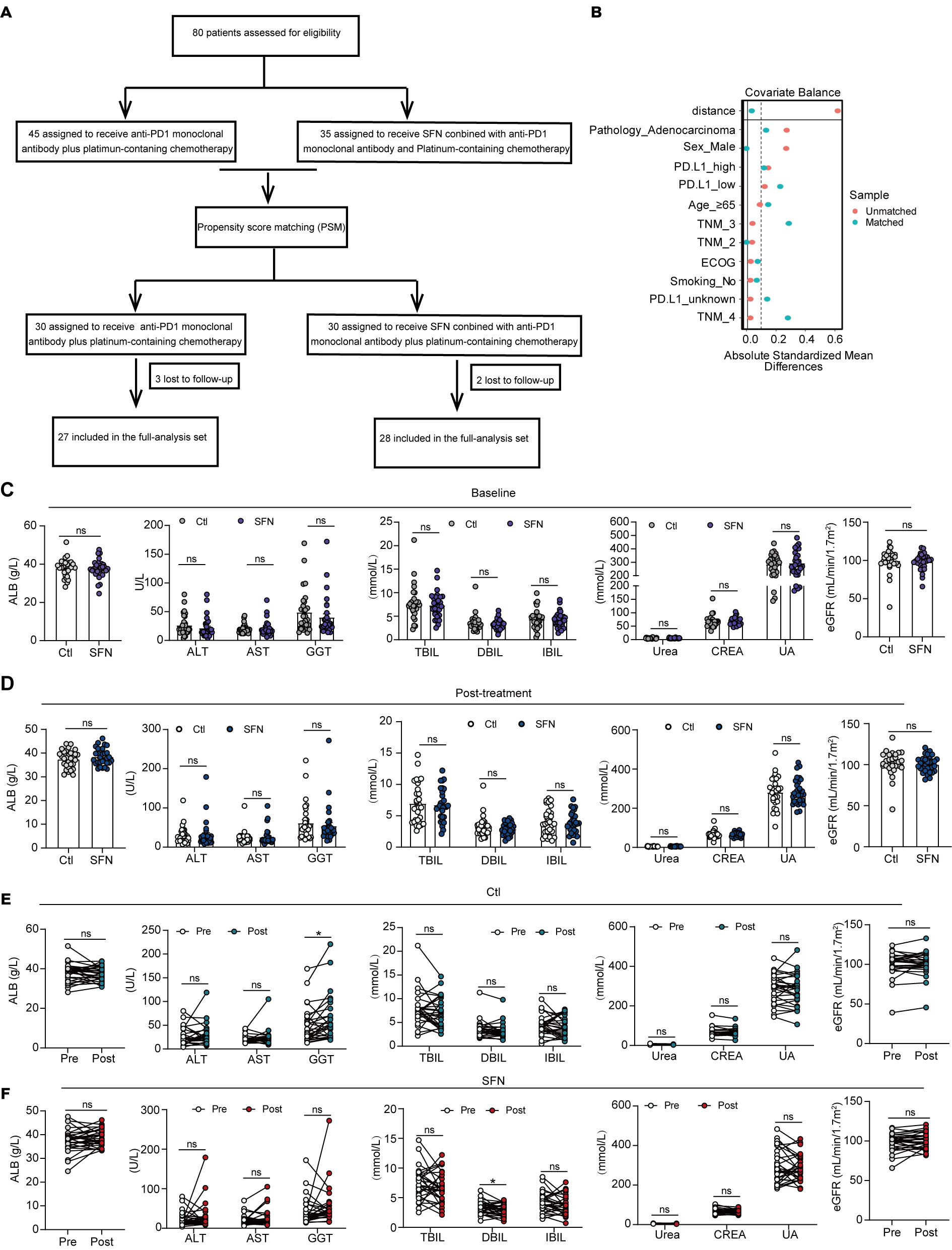
**

**Figure S1 Trial profile and safety analysis.** A. Eighty patients were included in the study. Of these, 45 patients were assigned to the control group and 35 to the experimental group. After propensity matching, each group comprised 30 patients. There were 3 cases lost to follow-up in the control group and 2 cases lost to follow-up in the experimental group. B. Propensity score matching. C-F. Liver and kidney function were analyzed before and after treatment.

**
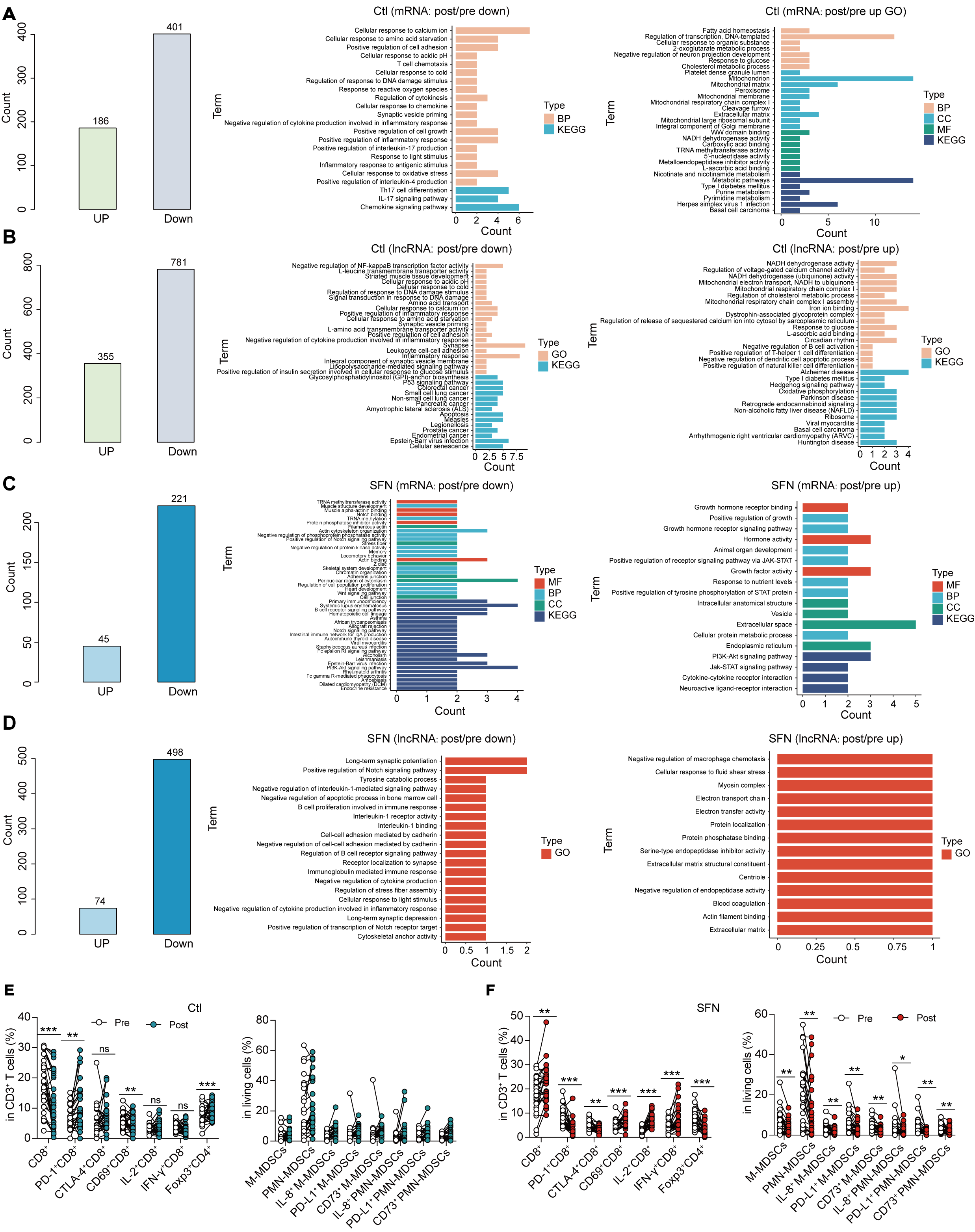
**

**Figure S2 SFN treatment enhance the anti-tumor response in peripheral.** Peripheral blood mononuclear cells were obtained from the blood of control and SFN treated patients at the baseline and after treatment. Then lncRNA-seq was performed. A. The different gene numbers of mRNA and GO/KEGG analysis were performed at the baseline and after treatment in control group. B. The different gene numbers of lncRNA and GO/KEGG analysis were performed at the baseline and after treatment in control group. C. The different gene numbers of mRNA and GO/KEGG analysis were performed at the baseline and after treatment in experimental group. D. The different gene numbers of lncRNA and GO/KEGG analysis were performed at the baseline and after treatment in experimental group. Flow cytometry was used to detect the CD8^+^ T cells and MDSCs at the baseline and after treatment in the control (E) and SFN (F) groups. (ns, non-significant difference, *p<0.05, **p<0.01, ***p<0.001).


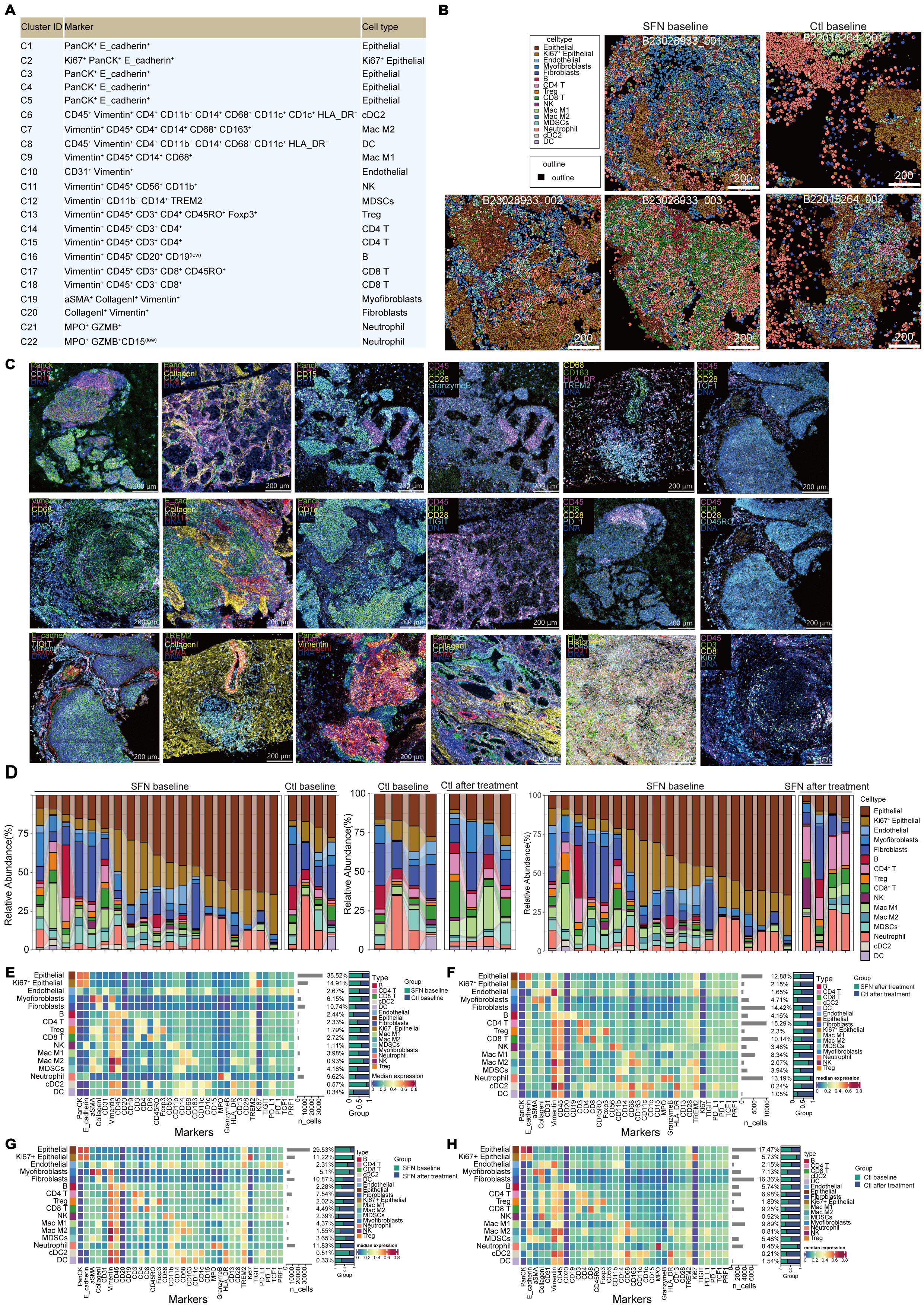


**Figure S3 IMC analysis of tumor microenvironment.** A-C. The antibody panel that used to stain the cancer tissue before and after treatment., with representative IMC images shown. D. Proportions of cell types. E. Heatmaps displaying the expression of markers in each cell type, along with the ratios of each cell type at the baseline in control and experimental groups. F. Heatmaps displaying the expression of markers in each cell type, along with the ratios of each cell type after treatment in control and experimental groups. G. Heatmaps displaying the expression of markers in each cell type, along with the ratios of each cell type before and after treatment in experimental groups. H. Heatmaps displaying the expression of markers in each cell type, along with the ratios of each cell type before and after treatment in control groups.


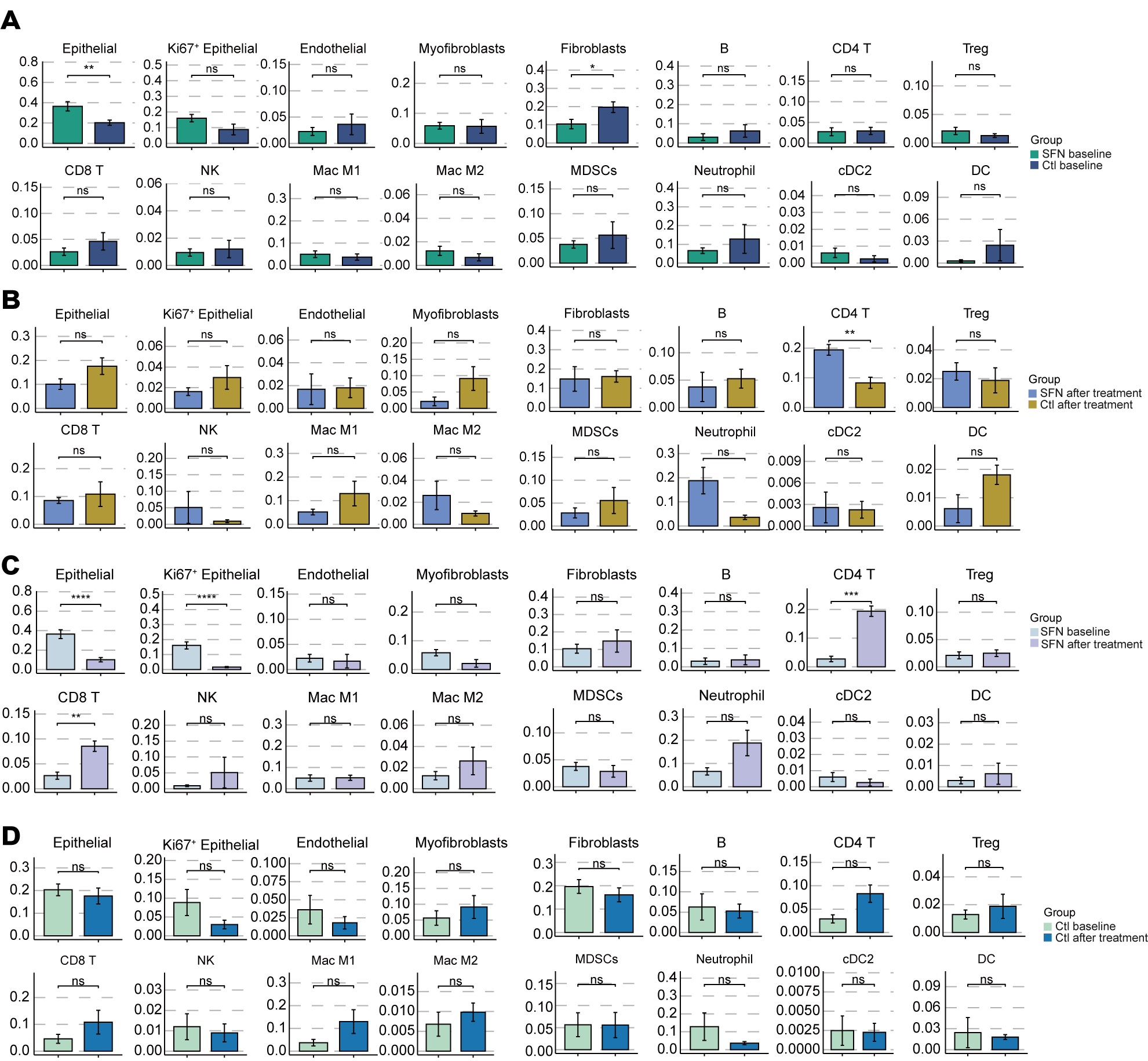


**Figure S4 The cell proportions difference was analyzed.** A. Boxplots depicting proportion distributions of key cell types at the baseline in control and experimental groups. B. Boxplots depicting proportion distributions of key cell types after treatment in control and experimental groups. C. Boxplots depicting proportion distributions of key cell types before and after treatment in experimental groups. D. Boxplots depicting proportion distributions of key cell types before and after treatment in control groups. (ns, non-significant difference, *p<0.05, **p<0.01, ***p<0.001).

**Table S1 Baseline characteristics of NSCLC patients with sequential and simultaneous combination before and after matching.**

| **Characteristics** | **Level** | **Before Matching** | | |  | **After Matching** | | |
| --- | --- | --- | --- | --- | --- | --- | --- | --- |
|  |  | **Con** | **Exp** | **SMD△** |  | **Con** | **Exp** | **SMD△** |
| n |  | 45 | 35 |  |  | 30 | 30 |  |
| Age (%) | <65 | 34 (75.6) | 25 (71.4) | -0.091 |  | 23 (76.7) | 21 (70.0) | -0.148 |
|  | ≥65 | 11 (24.4) | 10 (28.6) | 0.091 |  | 7 (23.3) | 9 (30.0) | 0.148 |
| Sex (%) | Female | 9 (20.0) | 4 (11.4) | -0.269 |  | 4 (13.3) | 4 (13.3) | 0.000 |
|  | Male | 36 (80.0) | 31 (88.6) | 0.269 |  | 26 (86.7) | 26 (86.7) | 0.000 |
| Smoking (%) | Yes | 29 (64.4) | 23 (65.7) | 0.027 |  | 21 (70.0) | 20 (66.7) | -0.070 |
|  | No | 16 (35.6) | 12 (34.3) | -0.027 |  | 9 (30.0) | 10 (33.3) | 0.070 |
| Pathology (%) | Squamous | 28 (62.2) | 17 (48.6) | -0.273 |  | 15 (50.0) | 17 (56.7) | 0.133 |
|  | Adenocarcinoma | 17 (37.8) | 18 (51.4) | 0.273 |  | 15 (50.0) | 13 (43.3) | -0.133 |
| TNM (%) | 2 | 1 (2.2) | 1 (2.9) | 0.038 |  | 1 (3.3) | 1 (3.3) | 0.000 |
|  | 3 | 15 (33.3) | 11 (31.4) | -0.041 |  | 7 (23.3) | 11 (36.7) | 0.287 |
|  | 4 | 29 (64.4) | 23 (65.7) | 0.027 |  | 22 (73.3) | 18 (60.0) | -0.281 |
| ECOG (%) | 0 | 34 (75.6) | 26 (74.3) | -0.029 |  | 23 (76.7) | 22 (73.3) | -0.076 |
|  | 1 | 11 (24.4) | 9 (25.7) | 0.029 |  | 7 (23.3) | 8 (26.7) | 0.076 |
| PD-L1 (%) | high | 2 (4.4) | 3 (8.6) | 0.147 |  | 2 (6.7) | 3 (10.0) | 0.119 |
|  | low | 14 (31.1) | 9 (25.7) | -0.123 |  | 12 (40.0) | 9 (30.0) | -0.229 |
|  | unknown | 29 (64.4) | 23 (65.7) | 0.027 |  | 16 (53.3) | 18 (60.0) | 0.140 |

**Table S2 Treatment-related adverse events and serious treatment-related adverse events in each group.**

|  | Control group  (n=30) | | SFN group  (n=30) | |
| --- | --- | --- | --- | --- |
|  | 1-2 | 3-4 | 1-2 | 3-4 |
| White blood cell count decreased | 8 | 4 | 19 | 4 |
| Neutrophil count decreased | 6 | 5 | 4 | 2 |
| Platelet count decreased | 6 | 3 | 8 | 5 |
| Anaemia | 29 | 0 | 24 | 4 |
| Nausea | 11 | 1 | 13 | 0 |
| Hepatic function abnormal | 20 | 0 | 13 | 0 |
| Myocardial injury | 3 | 0 | 4 | 0 |
| Asthenia | 7 | 0 | 10 | 0 |
| Hypothyroidism | 4 | 0 | 3 | 0 |
| Dyslipidemia | 0 | 0 | 6 | 0 |
| Numbness of extremities | 1 | 0 | 4 | 1 |
| Mucosal injury | 4 | 0 | 0 | 1 |

**Materials and methods**

**Study design and assessment**

The primary endpoints were PFS and OS. The main secondary endpoints were ORR, DCR, clinical benefit rate, and safety. Clinical responses for each target lesion were assessed every two cycles according to RECIST (version 1.1). Adverse events were recorded according to the Common Terminology Criteria for Adverse Events (CTCAE) version 5.0. All adverse events in treated patients were recorded until 3 months after the last anti-PD-1 antibody dose or death, whichever occurs later.

We enrolled patients at a ratio of 1.5:1, excluding those who had not completed the treatment cycle. Ultimately, 80 patients were included in the analysis. The control group consisted of 45 patients treated with chemotherapy combined with anti-PD-1 antibody. Besides platinum-based drugs, this regimen included pemetrexed, paclitaxel, docetaxel or gemcitabine. Blood samples were collected during the platinum-based treatment cycles. The experimental group (n=35) received SFN (100 mg/day, *per os*, d1-84: Pu'er Qiyun Biotechnology Co., LTD) plus chemo-anti-PD-1 combination therapy. This clinical trial was approved by the Clinical Research Ethics Committee of the First Affiliated Hospital of Zhengzhou University.

This clinical research involved the collection of data from patients treated with either control group or experimental group. The primary outcome was the effectiveness of each treatment, measured by relevant clinical indicators. Demographic and clinical characteristics of the patients, such as age, sex, smoking, pathology, TNM, ECOG and PD-L1, were collected. To reduce the impact of potential confounders, we employed propensity score matching. This statistical technique aimed to balance the covariates between the treatment groups, ensuring that any observed differences in the outcome were attributed to the treatments themselves rather than the influence of confounding factors.

The propensity scores were calculated using logistic regression, taking into account the aforementioned demographic and clinical characteristics. We performed 1:1 greedy nearest neighbor matching with a caliper of 0.3. The method functionally relied on the R package MatchIt. A distance was computed between unit of one group and another, and, one by one, each unit was assigned a control unit as a match. The matching was “greedy” in the sense that no action was taken to optimize an overall criterion; each match was selected without considering the other matches that might occur subsequently. After matching, we performed a comparison of clinical outcomes between the two groups using appropriate statistical tests.

**Cell lines**

LLC cell lines were purchased from the cell bank of the Chinese Academy of Sciences (Shanghai, China). The cells were cultured in DMEM high glucose (Sigma-Aldrich, St. Louis, MO, USA) containing10% FBS (Lonsera, Uruguay), 100 U/mL penicillin, and 100 μg/mL streptomycin without mycoplasma at 37°C in a humidified 5% CO_2_ incubator.

**Mice**

Female C57BL/6 mice (weight: 16-18 g, aged: 4-6 weeks) were obtained from the animal facility (Zhejiang Weitong Lihua Experiment Animal Technology) and were fed in the Animal Experiment Center of Henan Province. All animal related experiments were conducted accordin to the guideline of the Care and Use of Laboratory Animals. All animal studies were approved by the Institutional Animal Care and Use committee of the First Affiliated Hospital of Zhengzhou University.
